# A large-scale genetic screen identifies genes essential for motility in *Agrobacterium fabrum*

**DOI:** 10.1371/journal.pone.0279936

**Published:** 2023-01-04

**Authors:** Diana G. Calvopina-Chavez, Robyn E. Howarth, Audrey K. Memmott, Oscar H. Pech Gonzalez, Caleb B. Hafen, Kyson T. Jensen, Alex B. Benedict, Jessica D. Altman, Brittany S. Burnside, Justin S. Childs, Samuel W. Dallon, Alexa C. DeMarco, Kirsten C. Flindt, Sarah A. Grover, Elizabeth Heninger, Christina S. Iverson, Abigail K. Johnson, Jack B. Lopez, McKay A. Meinzer, Brook A. Moulder, Rebecca I. Moulton, Hyrum S. Russell, Tiana M. Scott, Yuka Shiobara, Mason D. Taylor, Kathryn E. Tippets, Kayla M. Vainerere, Isabella C. Von Wallwitz, Madison Wagley, Megumi S. Wiley, Naomi J. Young, Joel S. Griffitts

**Affiliations:** Department of Microbiology and Molecular Biology, Brigham Young University, Provo, UT, United States of America; Texas A&M University, UNITED STATES

## Abstract

The genetic and molecular basis of flagellar motility has been investigated for several decades, with innovative research strategies propelling advances at a steady pace. Furthermore, as the phenomenon is examined in diverse bacteria, new taxon-specific regulatory and structural features are being elucidated. Motility is also a straightforward bacterial phenotype that can allow undergraduate researchers to explore the palette of molecular genetic tools available to microbiologists. This study, driven primarily by undergraduate researchers, evaluated hundreds of flagellar motility mutants in the Gram-negative plant-associated bacterium *Agrobacterium fabrum*. The nearly saturating screen implicates a total of 37 genes in flagellar biosynthesis, including genes of previously unknown function.

## Introduction

Flagellar motility is widespread in Gram-positive and Gram-negative bacteria, with motility systems in the Gram-negative enteric species *Escherichia coli* and *Salmonella enterica* being particularly well characterized. The gram-negative flagellum consists of an envelope-integrated basal body that drives rotary motion of an external hook-filament complex. The basal body includes a hollow rod connected to a series of rings embedded in each of the three envelope layers (inner membrane, peptidoglycan, and outer membrane). The rotary motion of the rod is driven by the flagellar motor, which consists of the rotor component along with stator modules that are mechanically stabilized by their association with the peptidoglycan wall. Flagellar motion is powered by the movement of protons through the stator modules and this movement is translated to locomotion by connection of the flagellar rod to a flexible extracellular hook that in turn connects to a propulsive filament. The spinning filament generates thrust by either clockwise or counterclockwise rotation (depending on the organism), and directionality of cell movement is ultimately controlled by a discontinuous pattern of “runs” (propulsive activity) and “tumbles” (reorientation by pausing or reversing flagellar rotation). The switching between runs and tumbles is directed by a chemosensory network consisting of chemoreceptors and associated “Che” proteins that transduce signals from attractants and repellents to the flagellar apparatus [1,2].

Flagellar assembly in all bacteria is thought to be controlled by a cascade of regulatory events consisting of master transcriptional regulators and sensors of flagellar assembly status. For example, in the enteric bacteria, the FlhD4C2 heterohexamer [3,4] (Class I) is the master transcriptional regulator of Class II genes responsible for assembly of the hook-basal body (HBB) structure [5]. FliA, a sigma factor required for Class III gene expression, is synthesized along with Class II proteins, but is bound by the anti-sigma protein FliM until the HBB is competent to efflux substrates through its secretory system. FliM is among these substrates, and so formation of a complete HBB licenses FliA to go on and activate transcription of the late (Class III) genes [6,7]. Flagellar assembly and composition in the rhizobiaceae (which includes species of Rhizobium, Sinorhizobium, and Agrobacterium) has been shown to differ from the enteric paradigm in several respects: VisN and VisR (Class IA) possess the role of master regulators (presumably forming a heterodimeric LuxR-family transcriptional activator complex), Rem (an OmpR-like transcription factor) is activated by VisNR, and is thus considered Class IB [8–11]. Rem differs from canonical two-component response regulators given that it is functionally active in the absence of regulatory domain phosphorylation. Furthermore, it has substitutions in key aspartyl residues making it a member of the aspartate-less response regulator class [8,12]. Rem is directly responsible for transcriptional activation of Class II genes for HBB assembly. Rem activity may be modulated by inputs such as the motility inhibitor MirA [13]. The regulation of Class III genes has been under active investigation particularly in the Caulobacter system, where FlbT functions as a translational repressor in conjunction with a flagellin-like co-repressor; secretion of the flagellin-like co-repressor, which is potentiated by the secretion chaperone FlaF, leads to translational activation of Class III transcripts encoding motility factors such as flagellins and Che proteins [14,15].

Flagellar Motility in the *Rhizobiaceae* features several characteristics not encountered in the enteric model organisms. First, *Rhizobiaceae* tend to encode multiple flagellin proteins. In *A*. *fabrum*, four flagellin-encoding genes (*flaA*, *flaB*, *flaC*, and *flaD*) are found. Of these, only *flaA* is required for motility, while at least one of the remaining flagellin genes must be co-expressed with *flaA* for normal motility [16–18]. Second, the motor apparatus, which includes the highly conserved MotA and MotB stator proteins, are embellished in *Rhizobiaceae* by additional components such as MotC and MotE [19,20]. Finally, while enteric flagellar genes are widely distributed across the genome, the ~50 flagellar and chemotaxis genes in *Rhizobiaceae* are densely clustered in a region of roughly 50 kilobases on the circular chromosome [21]. Within this motility cluster, most of these genes are organized as polycistronic operons.

Here we report a large-scale transposon mutagenesis of *A*. *fabrum* and subsequent screen for motility-defective mutants. Nearly all of the responsible insertions fall within the motility cluster and highlight genes responsible for flagellar regulation and assembly, including several genes less well characterized or not previously assigned such functions. This work was carried out largely as a class project for a Brigham Young University microbial genetics course (MMBIO 360) involving 27 undergraduate students and two graduate teaching assistants.

## Materials and methods

### Strains and culture conditions

Strains used in this study are listed in S1 Table. The plasmid-cured *A*. *fabrum* strain UBAPF2 [22] was selected for streptomycin resistance by six independent student groups, resulting in motile starting strains BB01, YS01, BM01, IW01, CI01, and BL01. Donor *E*. *coli* strain DH5ɑ/pAB181 (D223) was used to deliver a mini-Tn5 transposon for mutagenesis, with helper strain DH5ɑ/pRK600 (B001) [23]. Routine culture of *A*. *fabrum* and *E*. *coli* was carried out in Luria broth (LB) containing (per liter) 10 g tryptone, 5 g yeast extract, 5 g NaCl and 1 ml of 2N NaOH, with 12 g of agar added to solidify when appropriate. Cultures were incubated at 37°C (*E*. *coli*) or 30°C (*A*. *fabrum*). Motility agar contained (per liter) 5 g tryptone, 2.5 g yeast extract, 0.5 g CaCl_2_ and 2 g of agar. For motility testing, cells were taken with wooden toothpicks, stabbed into motility agar, and allowed to incubate at 30°C for 2–3 days. For library enrichment (described below), motility agar was made instead with 1.7 g/L agar. Where appropriate, antibiotics were used as follows: streptomycin (Sm), 200 μg/ml; ampicillin (Ap), 100 μg/ml; chloramphenicol (Cm), 30 μg/ml; kanamycin (Km), 30 μg/ml; and neomycin (Nm), 100 μg/ml.

### Transposon mutagenesis and screening for motility defects

Bacterial conjugation by triparental mating was carried out by first growing the six initial Sm-resistant *A*. *fabrum* strains (the recipients), and donor and helper *E*. *coli* strains (for plasmids and strains details see S1 and S2 Tables) separately as patches on LB-agar with appropriate antibiotics. Cells were collected with toothpicks and suspended in liquid LB to equivalent levels of turbidity. Six matings (one for each recipient) were set up by combining 70 μl of each suspension, plating cell mixtures on LB-agar, and allowing overnight incubation. Resulting lawns were collected by suspending in LB containing 15% glycerol. Aliquots were stored at -80°C. Transposants were selected by plating mating suspensions on LB-agar containing Sm and Nm. From each of the six matings, approximately 2 x 10^5^ mutant colonies were selected. The selected libraries were collected in LB containing 15% glycerol and stored frozen as before.

For enrichment of non-motile *A*. *fabrum* mutants from each transposon library, 1 μl of suspension (containing approximately 1 x 10^7^ cells) was stabbed into 0.17% motility agar and allowed to incubate for 72 hours. At this point a clean toothpick was inserted into the original stab site to pick up cells that had not migrated into the motility agar. These cells (approximately 1 x 10^6^ per toothpick) were grown to saturation in LB-Sm/Nm, then combined with glycerol and stored as frozen aliquots for subsequent screening. For screening, the transposon libraries (enriched for non-motile mutants) were plated to single colonies on LB-Sm/Nm, and colonies were stabbed into motility agar one by one, with approximately 60 stabs per 100-mm plate. Non-motile clones were extracted and restreaked for further analysis to retest the phenotype and identify transposon insertion sites.

### Arbitrarily primed PCR and Sanger sequencing

In the first round of Arbitrary (Arb) PCR, 1.5 μl of lysed, boiled cells from each strain was added to 15.4 μl of water, 2 μl of Taq buffer, 0.5 μl 10 mM dNTP, 0.15 μl Taq, 0.15 μl of 100 μM of forward primer (2100), and 0.3 μl of 100 μM reverse primer (2102 or 2103). PCR was carried out under the following conditions: after initial denaturation (94°C for 1 min), cycling 6 times (94°C for 15 sec, 33°C for 45 sec, 70°C for 45 sec), and cycling 30 times (94°C for 15 sec, 43°C for 30 sec, 70°C for 45 sec). In the second round of Arb-PCR, 0.9 μl of the amplified DNA from the first round of PCR was added to 16.1 μl of water, 2 μl of Taq buffer, 0.5 μl of 10 mM dNTP, 0.15 μl Taq, 0.15 μl of 100 μM forward primer (2101) and 0.15 μl of 100 μM reverse primer 2104. The second round of PCR was carried out under the following conditions: after initial denaturation (94°C for 1 min), cycling 30 times (94°C for 15 sec, 55°C for 15 sec, 70°C for 45 sec). Sanger Sequencing was carried out on the amplified PCR products to identify transposon insertion sites. The first-round arbitrary primer 2102 was normally used, but in cases of unacceptable product or low-quality sequence for a given mutant, the alternative primer 2103 was used (see S3 Table for primer sequences).

From the data obtained from Sanger Sequencing, the …GAGACAG sequence at the end of the mini-transposon was located and the 30 nucleotides following this were used to find the position in the *A*. *fabrum* genome, using BLASTN (in GenBank accession AE007869.2) [24]. In each case, the transposon insertion location, directionality, and identity of the disrupted gene was noted (given in S1 and S2 Files). Where appropriate, the corresponding protein sequences were analyzed bioinformatically using BLASTP, signalP, and Pfam [25,26]. A manual annotation of the *A*. *fabrum* motility gene cluster was also carried out using BLASTP (given in S3 File) [24].

### Construction of strains with in-frame deletions

Plasmids that contained homology regions for each target gene were created. Homology regions were designed to contain 300bp of DNA on each side of the gene of interest, maintaining the first and last several codons for that gene. Sequences for each target gene were retrieved from GenBank accession number AE007869.2. Parent plasmid pJG1108 containing XbaI-SalI-*gus*-*sacB*-*kanR* was digested with XbaI and SalI. Inserts were amplified from BB01 DNA using primers listed in S3 Table and prepared for 3-way ligations in which the joint between right and left homology regions is the 6-base sequence CCCGGG (XmaI, encoding Pro-Gly). Ligation products were transformed into *E*. *coli* DH5α and sequence verified. Deletion plasmids were transferred into *A*. *fabrum* strain BB01 by triparental mating, as described above for transposon mutagenesis and transconjugant clones were selected on LB-SmNm. Four individual colonies from each mating were carried over to selection on LB containing sucrose and X-Gluc (5-Bromo-4-chloro-3-indoxyl-beta-D-glucuronide cyclohexylammonium salt) (100 μg/mL). After 72 hours, cells from white colonies were suspended in PCR lysis buffer (5mM Tris pH 8.0, 2mM EDTA, 0.5% Triton X-100) and heated to 95°C for 5 minutes. Confirmatory PCR was carried out with primers listed in S3 Table. Successful deletion was verified by band down-shift on an agarose gel. Motility tests for deletion strains were carried out as described above.

### Construction of plasmids for complementing knock-out strains

For complementing mutants Δ*ATU0568*, Δ*ATU0583*, Δ*flgN*, and Δ*motF* in *A*. *fabrum*, plasmid pKJ056 was used (S1A Fig). This plasmid allows expression of downstream genes by read-through transcription of the kanamycin-resistance (*kanR*) gene. All of the genes to be complemented were amplified from the *A*. *fabrum* C58 genome with their respective primers (S3 Table). pKJ056 was digested with EcoRI and BamHI as were all the amplified products and then ligated together. Ligated plasmids were transformed into *E*. *coli* DH5α and sequence verified. For *visNR* complementation experiments, three replicative plasmids were created that contain *visN*, *visR*, or *visNR* under the control of the P_vis_ promoter. Parent plasmid pPG012 containing BamHI-XbaI-*kanR* was digested with BamHI and XbaI (S1B Fig). Inserts were amplified from strain C237 with primers listed in S3 Table. Ligation products were transformed into DH5α and sequence verified. All complementation plasmids were transferred into their respective knock-out strains by triparental mating as described above for transposon mutagenesis. Motility tests were carried out as described above. Complete DNA sequences of parent plasmids are provided in S4 File.

## Results

### An enrichment-aided screen for motility mutants in *A*. *fabrum*

A screen for motility mutants in *A*. *fabrum* was carried out by first mutagenizing Sm-resistant derivatives of the plasmid-cured strain UBAPF2 [22]. This strain imposes reduced environmental hazard for use in an undergraduate lab, as it is unable to infect plants, and its genome is somewhat reduced without loss of motility, increasing the probability of finding non-motile mutants in a forward genetic screen. Mutagenesis by triparental mating with the donor strain DH5ɑ/pAB181 (S2A and S2B Fig) resulted in over 10^6^ Sm/Nm-resistant transposants as a starting population for screening. Given the proportion of the genome expected to control flagellar motility (around 0.8%, or 40–50 genes), we anticipated the need to assay approximately 50,000 transposants in order to approach saturation. Rather than use this approach, we pre-enriched the mutant population for non-motile cells S2C Fig. This was done by first injecting motility agar with the mutant population and then allowing cells to swim away from the site of inoculation for several days. The sub-population remaining at the site of injection was then recovered in a manner that avoided “bottlenecking” effects (see Methods). This enrichment procedure was carried out across multiple plates from six independent transposon libraries to ensure the maintenance of genetic diversity in the enriched libraries.

**Fig 1:**
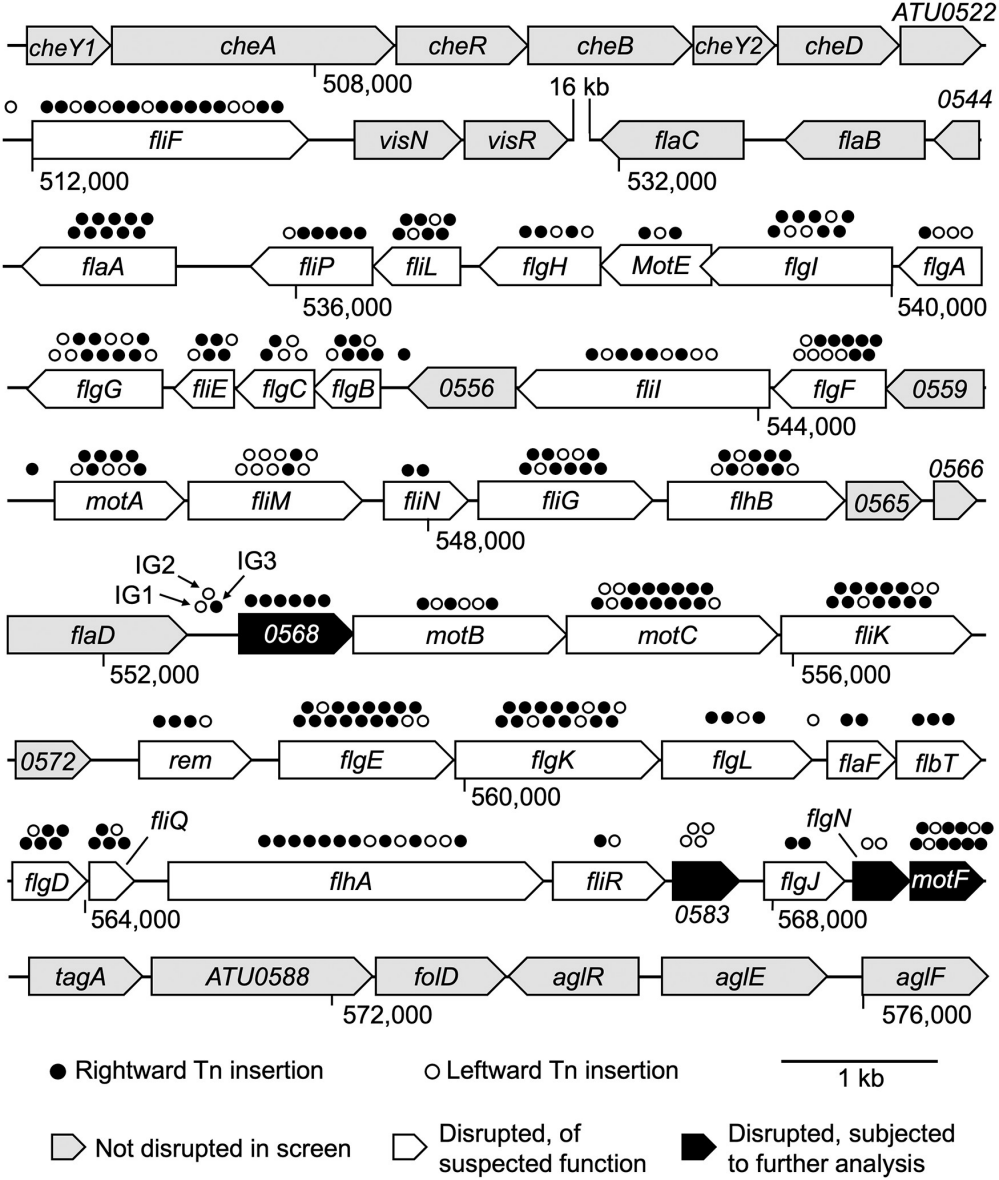
Annotated motility cluster in *A*. *fabrum*. Shown in gray are genes that were not disrupted in the screen. Shown in white are genes that were disrupted in the screen and are of strongly suspected function. Shown in black are genes that were disrupted in the screen and were subjected to further analysis. White dots on top of the genes show that a transposon landed in rightward orientation (referring to *kanR* transcription), while black dots indicate that a transposon landed in leftward orientation. A 16-kb region not shown in the figure includes genes that are not predicted to be involved in flagellar assembly, and no genes in this region were disrupted in the screen.

Libraries enriched for non-motile mutants were plated to single colonies, and these were screened colony-by-colony for motility defects. Only strongly non-motile mutants were carried forward in our analysis. With the enrichment strategy described above, over 25% of colonies assayed were strongly defective in motility (S2 Fig). Around 500 such mutants were streaked to isolation and retested for motility. Of these, 360 confirmed mutants were used to map transposon insertion sites by arbitrarily primed PCR. This analysis revealed 314 unique insertions possibly associated with the motility defect. Most of these (301 insertions) were confined to a known cluster of flagellar motility genes, comprising nucleotides (512,099–569,457) in the *A*. *fabrum* genome (GenBank Accession AE007869.2, and S1 File). The remaining 13 insertions were distributed across the genome (S2 File). Remarkably, genes disrupted outside the motility cluster were always represented by only a single insertion, while genes disrupted inside the motility cluster (Fig 1) were always represented by at least two independent insertions (an average of 8 insertions per gene). Therefore, we have focused our follow-up analysis on genes within the cluster.

### A ‘parts list’ for *A*. *fabrum* flagellar motility that includes previously undescribed genes

**Fig 2:**
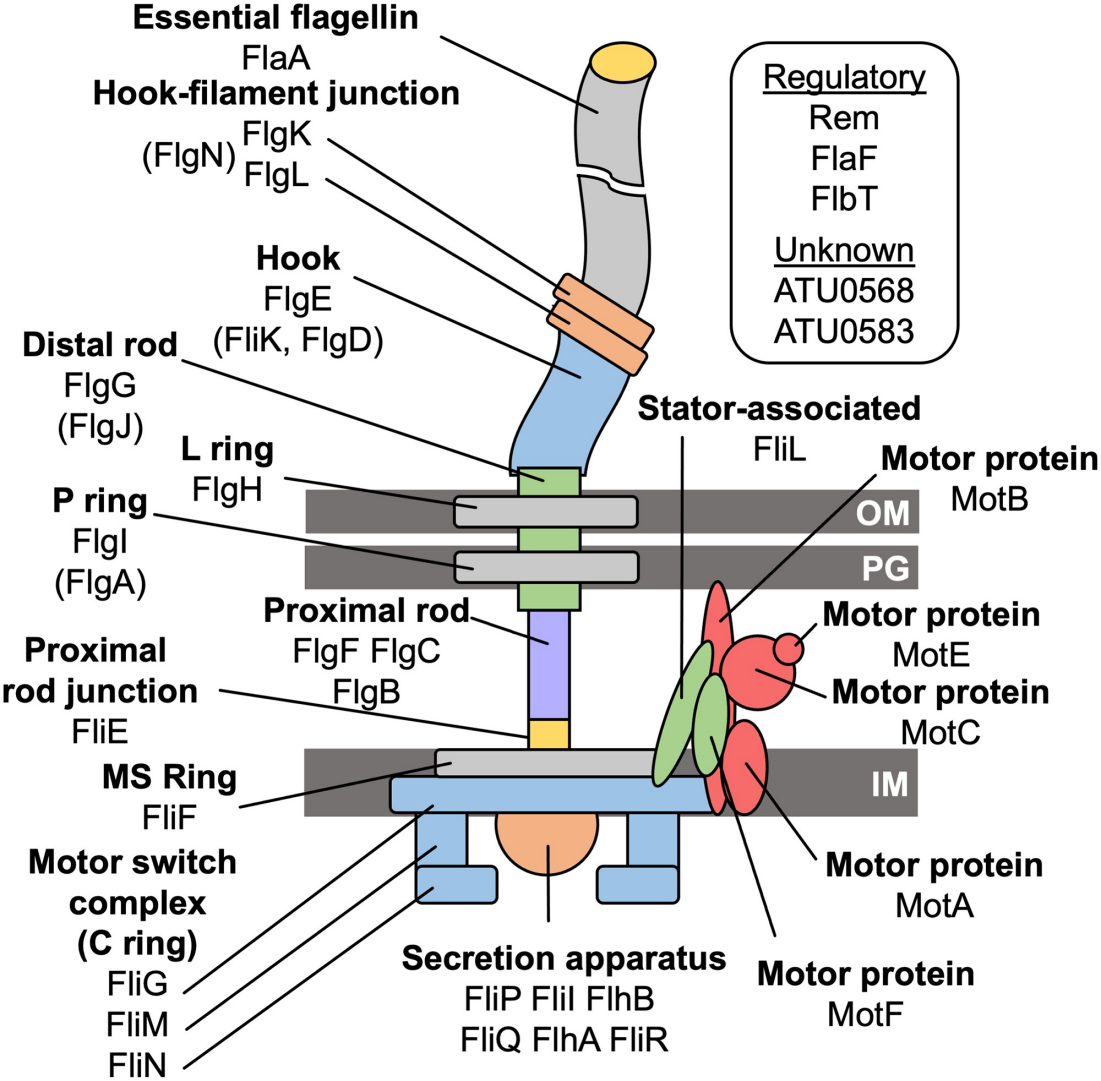
The putative structure of the *A*. *fabrum* flagellum and the proteins from which it is constituted. The diagram includes the orientation of the flagellum with respect to major cell envelope components: Outer membrane (OM), peptidoglycan (PG), and the inner membrane (IM). Also labeled are the main functional units of the flagellum and the specific proteins from which they are made, all of which were implicated in this genetic screen. Proteins with regulatory or unknown functions are listed in the box.

Genes hit in our screen represent 37 proteins: 33 with known or suspected functions. At the time this screen was carried out, four of these proteins were of unknown function. Most of these proteins map to specific components of a model Gram-negative bacterial flagellum, as depicted in Fig 2. These components include the flagellar secretion apparatus (FliP, FliI, FlhB, FliQ, FlhA, FliR), the cytoplasmically localized C ring (FliM, FliN, FliG), the inner membrane-localized MS ring (FliF), the proximal rod junction (FliE), the proximal rod (FlgB, FlgC, FlgF), the P ring (FlgI), the L ring (FlgH), the distal rod (FlgG), core stator proteins (MotA, MotB), stator-associated proteins (MotC, MotE, FliL), hook (FlgE), hook-filament junction (FlgK, FlgL), and filament (FlaA). Other genes disrupted in the screen encode proteins that may play transient roles in directing flagellar assembly, including the rod capping protein (FlgJ), the hook capping protein (FlgD), the hook length regulator (FliK), and the P ring assembly chaperone (FlgA). Disrupted genes with transcriptional or translational regulatory functions include the Class IB transcriptional regulator Rem, and the functionally coupled translational regulators FlbT and FlaF.

At the time this screen and analysis were carried out, four genes were of unknown function, and they were subjected to further analysis. These genes were designated *ATU0568*, *ATU0583*, *ATU0585*, and *ATU8132* (according to the naming system in GenBank accession AE007869.2, and see Fig 1). A BLASTP search revealed that ATU0568 contains a DUF4231 domain from the SLATT superfamily, which contains a pair of N-terminal transmembrane helices and a helical C-terminal cytoplasmic region [27]. A Pfam search of ATU0568 did not yield any homology or domain similarities to previously characterized proteins [26]. Bioinformatic analysis for ATU0583 suggested that this protein does not contain a signal peptide, nor does it contain any domain similarities to known proteins. A BLASTP of ATU0585 did not yield any regions of local similarities to previously characterized proteins. ATU0585 is not predicted to have a signal peptide according to signalP, and Pfam analysis of this protein did not yield any domain similarities to known proteins [25,26]. A BLASTP search of ATU8132 indicated that this protein shares homology with a FliL superfamily domain in *Agrobacterium fabrum*. SignalP predicted a potential signal peptide in ATU8132 suggesting that this protein is not localized in the cytoplasm, but it could be membrane localized or secreted. Pfam analysis revealed that the N-terminal residues of ATU8132 (aa 4–12) are predicted to be a hydrophobic region of a signal peptide, while aa 18–177 contain a membrane-bound region and reside on the outside of the membrane (in the periplasm or extracellular region).

**Fig 3:**
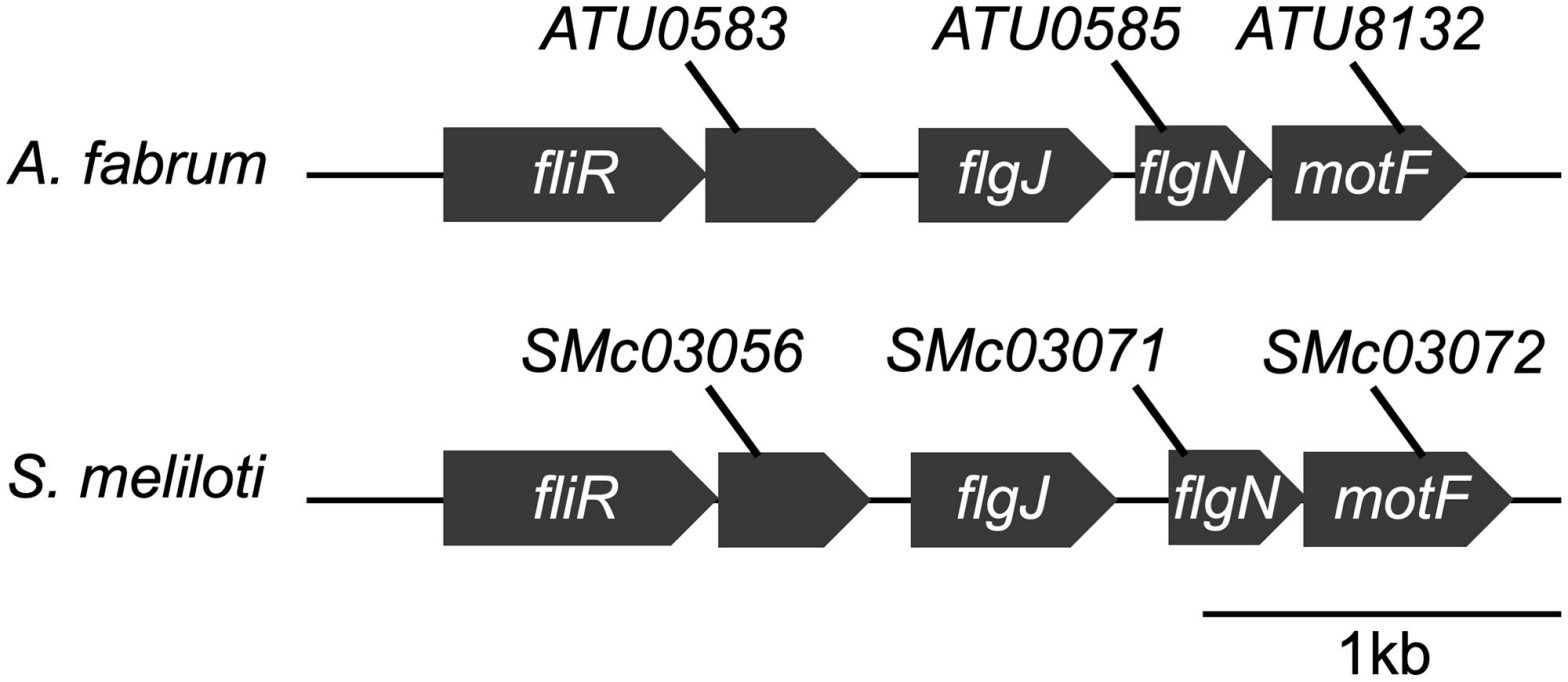
Synteny of four gene cluster required for motility in *Sinorhizobium meliloti* and *Agrobacterium fabrum*. *ATU0585* was renamed *flgN* and *ATU8132* was renamed *motF*.

A recent study by Sobe et al. [28] revealed a four-gene cluster that is required for motility in *Sinorhizobium meliloti* (*S*. *meliloti*) strain RU11/001. This cluster in *S*. *meliloti* (*SMc03056*-*SMc03071*-*SMc03072*-*SMc03057*) shows high synteny to an analogous region in *A*. *fabrum* shown in this study (*ATU0583*-*flgJ*-*ATU0585*-*ATU8132*) (Fig 3). Their study reveals that absence of the *S*. *meliloti* orthologs of *ATU0583*, *flgJ*, and *ATU0585* result in the absence of FlaA flagellin production. This is consistent with these three genes being involved at an earlier stage of flagellar biosynthesis [9]. By homology, this suggests that ATU0583, FlgJ (*A*. *fabrum*), and ATU0585 also function prior to flagellin secretion. A multiple sequence alignment of ATU0585, Smc03072, and FlgN (*Salmonella enterica*) reveals several identical amino acids justifying renaming ATU0585 as FlgN. The S. *meliloti* study also revealed that mutants lacking the *S*. *meliloti* ortholog of *ATU8132* were able to produce FlaA at levels comparable to wild-type, consistent with a role in a late stage of flagellar assembly or motor function and renamed it *motF*. In this study, *ATU8132* has also been renamed *motF*.

**Fig 4:**
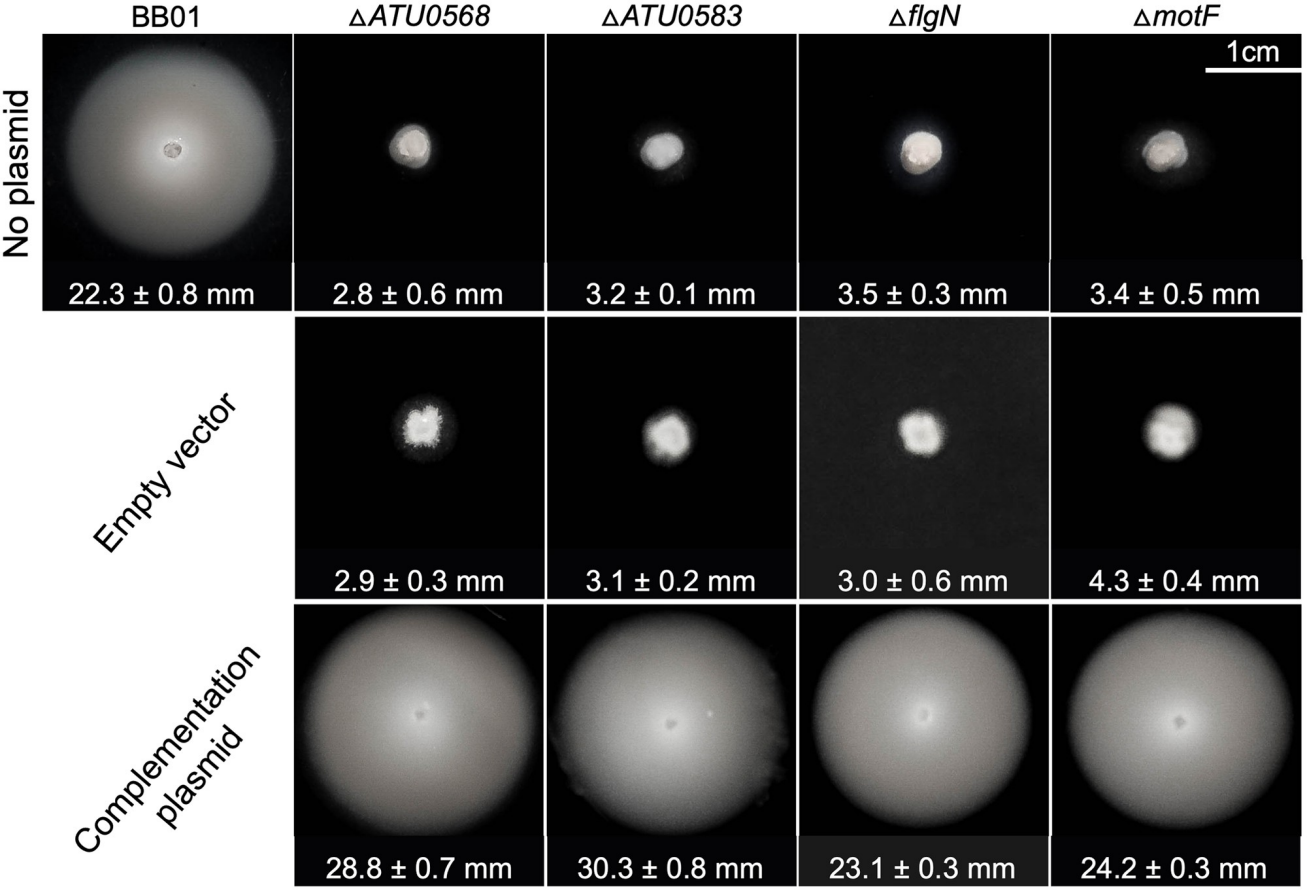
In-frame deletions and complementation experiments of the four genes subjected to this study. Motility assay for strains with each in-frame deletion and their corresponding complementation assays. Swim rings were imaged 48 h after inoculation. Shown are the averages of swim ring diameter for four replicates per strain in millimeters (mm) and standard deviation from the mean. Statistical analysis is shown in S3 and S4 Figs. Strategy used for in-frame deletions is depicted in S5A Fig.

The four focal genes discussed above (*ATU0568*, *ATU0583*, *flgN*, *motF*) were deleted in a manner intended to eliminate possible polar effects. This was done by removing most of the gene but retaining the first and last several codons of each coding sequence. We refer to these as “in-frame deletion” strains. All four of these strains exhibited motility defects similar to those of the rest of the motility mutants identified in the transposon screen. These deletion strains could be restored to normal motility by complementation plasmids constitutively expressing the corresponding genes (Fig 4).

### Intergenic insertions associated with motility defects

Several insertions associated with motility defects occurred in intergenic regions within the motility cluster (see Fig 1). These are generally interpreted to result in mis-expression of a nearby gene (by disrupting a promoter, overexpression, or antisense RNA expression). It was less straightforward to explain the three intergenic insertions occurring between the genes *flaD* and *ATU0568*. As discussed below, *flaD* is not required for motility, though *ATU0568* is. These “*flaD*-*ATU0568*” intergenic insertions are intriguing because they occur over a region of 161 bp, with the two insertions farthest from *ATU0568* (called IG1 and IG2) transcribing toward *flaD* (see Fig 1). There is an alternative start codon located 69 bp upstream of the annotated start codon of *ATU0568*. However, this is not predicted to be disrupted by either insertion (IG1 and IG2) since the closest insertion (IG2) is located 105 bp upstream of this alternative start codon. Sequence specific details of this intergenic region are provided in S5B Fig. Within this region, there is also a predicted open reading frame in the reverse orientation that slightly overlaps with *ATU0568* that could explain this motility phenotype. To investigate whether an unannotated gene exists in this region, the sequence disrupted in IG1 and IG2 was manipulated by deleting 10 bp and replacing it with a 6-bp XmaI sequence. This changes the frame and sequence in this region. These two deletion strains exhibited normal motility (S6 Fig), suggesting that something specific to the original transposon insertions, which was not recapitulated by the 10-bp deletions, contributed to the motility defect.

### Suppressibility of the Δ*motF* motility phenotype

**Fig 5:**
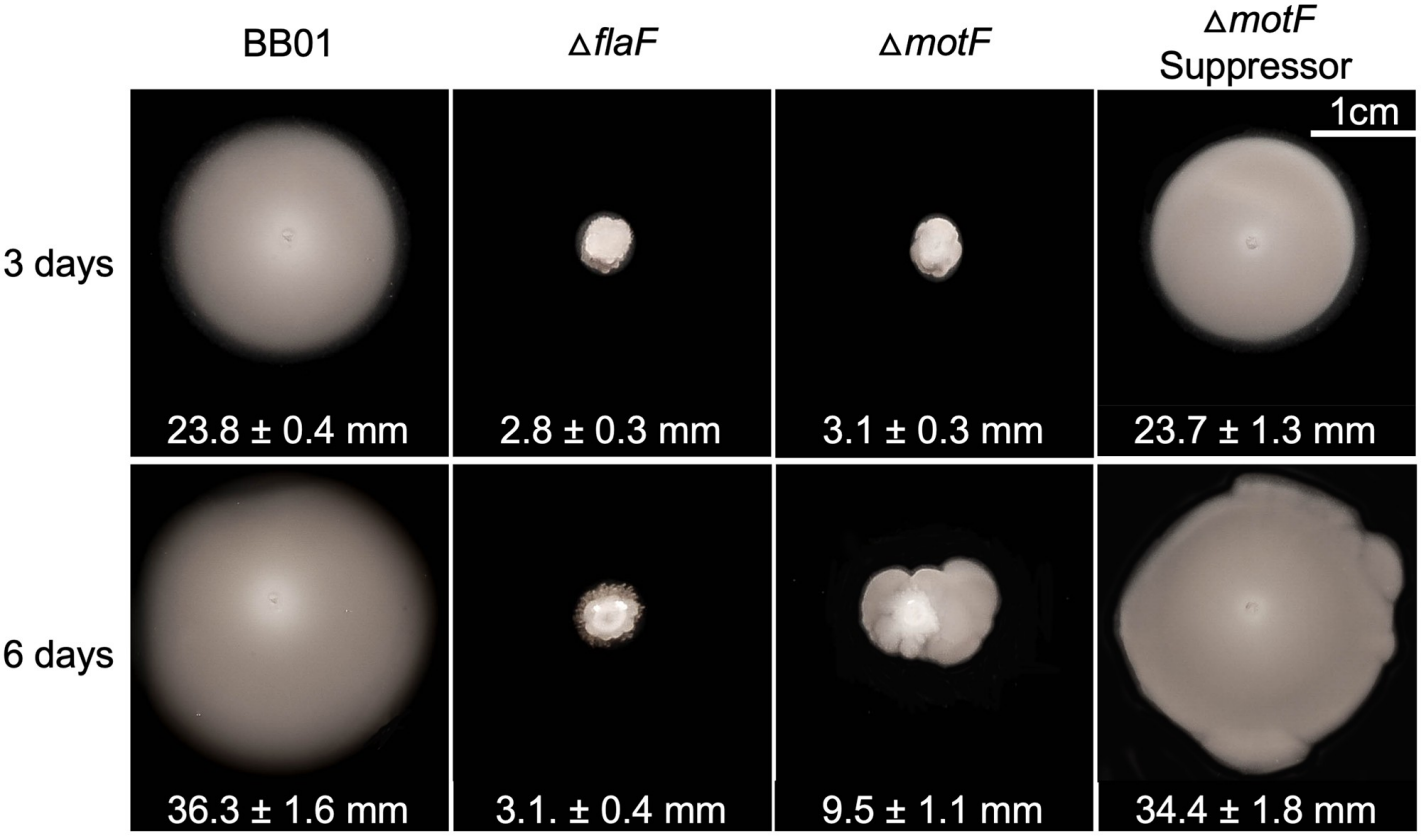
Δ*motF* has a suppressible phenotype. Strains were inoculated and allowed to swim for 3 (top) or 6 (bottom) days. The Δ*flaF* strain serves as a non-suppressible control. Below each strain is the average of swim ring diameters in millimeters (mm) for four replicates per strain and the standard deviation from the mean. Statistical analysis is shown in S7 Fig.

To begin exploring the functions of the four genes analyzed in this study (*ATU0568*, *ATU0583*, *flgN*, and *motF*), we used the in-frame deletion strains to test whether the mutant phenotype could be reversed by intergenic suppression. Genetic suppression was only observed for the Δ*motF* strain. This suppression phenomenon is shown in Fig 5, where extended cultivation of Δ*motF* in motility agar results in a blebbing pattern not seen in a non-suppressible mutant such as Δ*flaF*. Clones extracted from these zones of resumed motility exhibit near-wild-type motility upon retesting. Unlike wild-type cells, however, these suppressor strains seem to generate hypermotile derivatives, as evidenced by a blebbing pattern around the normal ring of motile cells. Future investigation to determine the molecular basis of Δ*motF* suppressibility will allow us to make connections between this new gene and previously studied motility functions. For instance, the study by Sobe et al. [28] reported a suppressor phenotype in strains lacking *motF*, in which suppressors regained partial motility (25%). They performed whole-genome sequence and found that all five suppressor strains contained mutations that mapped to the coding region of *motA* (G136S and Y248H).

### Analysis of master regulators *visN* and *visR*

**Fig 6:**
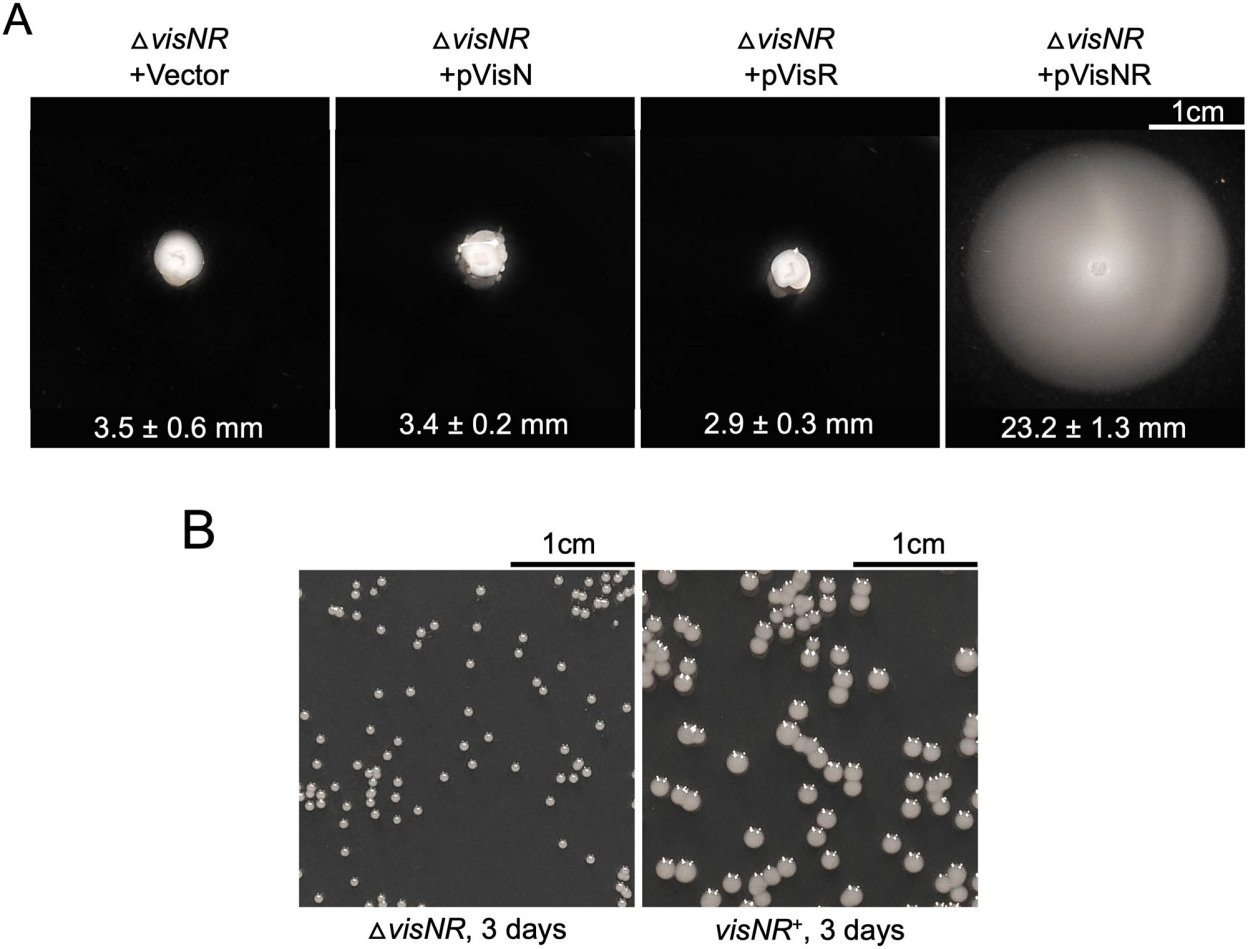
Characterization of the Δ*visNR* deletion strain. (A) Complementation test in which Δ*visNR* strains harbor plasmids indicated in S1 Fig. Shown are the averages of swim ring diameters in millimeters (mm) in four replicates per strain and standard deviation from the mean. Statistical analysis is shown in S9 Fig. (B) The left image shows a plate with Δ*visNR* colonies, and the right image shows a plate with parent strain BB01. Both strains were grown under the same conditions and images are shown at the same scale.

A readily noticeable discrepancy between our screening data (Fig 1) and known flagellar motility pathways in *Rhizobiaceae* is the absence of any mutants in the master regulator genes *visN* and *visR*. These two genes are adjacent to one another and encode a likely dimeric LuxR family transcriptional regulator required for motility in several *Rhizobiaceae* species including *S*. *meliloti* and *A*. *fabrum* [9]. The absence of insertions in the *visNR* operon is particularly striking given that the *fliF* gene upstream (which is roughly the same size as *visNR*) was disrupted by 18 different insertions. We have considered several explanations for this discrepancy, including the possibility that *visN* and *visR* have partially redundant functions in *A*. *fabrum*. To test this hypothesis, the *visNR* locus was removed from the motile strain BB01 and then modified with four complementation plasmid derivatives: pPG012 (vector-only control), pKJ121 (*visN* expression plasmid), pKJ122 (*visR* expression plasmid) and pKJ120 (*visNR* expression plasmid). This complementation analysis indicates that *visN* and *visR* are non-redundant: neither alone can restore motility to the Δ*visNR* strain; but co-expression of *visNR* completely restores motility (Fig 6A). This is consistent with results previously reported by Xu et al. [29]. The second hypothesis posits that *visN* and *visR* mutants did not arise in the screen because these mutants are hyper-adherent to neighboring cells. This property has been documented previously [29]. To test this, the Δ*visNR* mutant was mixed with BB01 that had been modified to constitutively express lacZ. Strong cell-cell adherence occurring in the mixed culture would result in sectored colonies upon plating on medium with the beta-galactosidase indicator X-gal (5-Bromo-4-Chloro-3-Indolyl β-D-Galactopyranoside). Sectored colonies were not observed in this analysis (S8 Fig). However, we observed that the *visNR*+ colonies are substantially larger than Δ*visNR* colonies (Fig 6B), presumably due to *visNR* being important for secretion of exopolysaccharides that are normally produced in abundance by wild-type cells [29]. Fig 6B shows colonies that had been incubated for 3 days, whereas during the screening process colonies were incubated for 2 days. From this, we presume that students may have systematically been biased against *visN* or *visR* mutants due to their unusually small colony morphology.

## Discussion

In this study, we carried out a comprehensive forward genetic screen for motility mutants in *Agrobacterium fabrum* in which 37 genes were identified as being required for motility based on strong loss-of-motility phenotypes. Based on the suspected functions of proteins encoded by these genes, nearly every molecular component generally required for flagellar assembly in Gram-negative bacteria was identified, in addition to several Alphaproteobacteria-specific functions and four proteins less well characterized. These four proteins (ATU0568, ATU0583, FlgN, and MotF) are all found within a 16-kb region on the right side of the motility gene cluster [30]. Based on database searches, these proteins do not appear in organisms outside of Alphaproteobacteria, suggesting they are specialized features within this class, and their presence almost exclusively in the family Rhizobiaceae suggests a particularly specialized role in these largely plant-associated bacteria. The Δ*motF* strain can spontaneously mutate to generate suppressor strains with restored motility (the other three mutants do not have this tendency).

Of the four flagellar filament genes in *A*. *fabrum* (*flaA*, *flaB*, *flaC*, and *flaD*), only *flaA* was identified in this screen, which was expected based on previous work showing that this is the only required flagellin, with the others serving subsidiary functions. Earlier studies have shown that Δ*flaA* mutants form straight flagellar filaments that result in very slow tumbling motion [18]. It appears that FlaA incorporates a functionally crucial helical attribute into the flagellar filament. A key residue in FlaA distinguishing it from the subsidiary flagellins is an Asn residue at position 129 that plays a role in establishing this helical property. It has been shown, however, that FlaA must function with at least one of the three subsidiary flagellins [16–18].

Three of our transposon mutants had insertions in three unique locations between the genes *flaD* and *ATU0568*. Within this intergenic region of 279 bp, they were located at position 84, 105 and 245. The transcription from the transposon read to the left for insertions at 84 and 105 and right for insertion 245. With these insertions spread so broadly across this region and the upstream flanking gene (*flaD*) not required for motility, we can only speculate how these transposon insertions bring about loss of motility. For two of these (IG1 and IG2), disruption of a 10-bp segment corresponding to the wild-type sequence, did not noticeably affect motility. We suspect that these intergenic insertions may disrupt an unusually large regulatory region upstream of *ATU0568* or may disrupt or mis-regulate some independent and unannotated feature required for motility.

Mutagenesis by transposon insertion can have polar effects on polycistronic operons. In this screen, with modest transcription emanating from the *kanR* gene of the transposon into the genome, we did not expect polar effects when the transposon was transcribed inserted in the same direction of the disrupted gene; but we expected possible polar effects when the transposon was transcribed in the opposite direction of the gene. We also observed that there was a strong directionality bias for transposon insertion in some genes such as *flaA* and *ATU0568*, but for most genes implicated in the study, insertions in both directions could be found.

The regulatory genes *rem*, *flbT*, and *flaF* were all hit in this screen. The Rem protein is a Class I transcriptional regulator that activates the expression of Class II structural and regulatory genes [8]. Two of these regulatory genes (*flbT* and *flaF)* are highly conserved among Alphaproteobacteria. In *Brucella melitensis*, FlbT acts as a translational activator for the synthesis of flagellin [31]. Like *flbT*, *flaF* is also conserved among Alphaproteobacteria, and is generally located upstream and is cotranscribed with *flbT* [15,32,33]. The *visN* and *visR* genes were not hit in this screen despite being essential for motility as top-level master transcriptional regulators [9,10,29]. As previous studies have shown, VisN and VisR not only positively regulate flagellar synthesis, but are also negative regulators of unipolar polysaccharide (UPP) synthesis and positive regulators of *exo* genes that control succinoglycan biosynthesis [29,34,35]. Δ*visNR* mutants are substantially smaller than wild type presumably due to decreased succinoglycan synthesis and more dry than wild type due to increased cellulose and UPP production [29]. We hypothesize that Δ*visNR* mutant colonies may not have been selected by the student researchers carrying out this screen due to their unusually small colony morphology.

The *Agrobacterium fabrum* C58 genome encodes roughly 20 methyl-accepting chemotaxis protein (MCP) homologs, and 9 *che* genes for chemotactic control of flagellar activity [30,36,37]. No *che* or *mcp* genes were hit in this screen, likely because we focused on mutants with strong non-swimmer phenotypes; mutants lacking *che/mcp* genes show significant but reduced motility [38]. A screen for mutants with partial loss of motility would surely point to chemotaxis functions, as well as other pathways contributing in more subtle ways to flagellar assembly and control.

## Supporting information

S1 Fig Parent plasmids used for complementation experiments. (A) Diagram of parent vector used to make complementation plasmids for Δ*ATU0568*, Δ*ATU0583*, Δ*flgN*, and Δ*motF* strains. Each of the four genes were constitutively expressed by read-through transcription of the *kanR* gene. (B) Plasmid used as parent vector for *visNR* complementation derivates. *visN*, *visR*, and *visNR* labels show where each gene was inserted; transcription of *visN*, *visR*, and *visNR* is driven by the native Pvis promoter.
(TIF)

S2 Fig A transposon screen for motility mutants in *A*. *fabrum* aided by pre-enrichment. (A) Transposon delivery plasmid used to carry out the mutagenesis. TE labels show the location of the repeated transposon elements. (B) Triparental mating scheme involving helper plasmid pRK600 and *A*. *fabrum* recipient strain UBAPF2 (SmR). (C) Enrichment strategy enabling high-efficiency screening for motility mutations.
(TIF)

S3 Fig Quantification of swim ring diameter of knock-out strains shown in Fig 4. BB01 is the wild-type control. Values are the averages of four swim rings per strain. Error bars show standard deviation from the mean. Different letters denote statistically significant differences (P < 0.05) according to Tukey multiple comparison test.
(TIF)

S4 Fig Quantification of swim ring diameter of the motility complementation test shown in Fig 4. Values are the averages of four replicates per strain. Error bars show standard deviation from the mean. Different letters denote statistically significant differences (P < 0.05) according to Tukey multiple comparison test.
(TIF)

S5 Fig Double-crossover strategy used for targeted in-frame deletions and sequence specific details of the *flaD*-*ATU0568* intergenic region. (A) The allelic replacement strategy used to construct deletion strains, using a plasmid that allows positive selection (*kanR*), negative selection (*sacB*), and color detection (*gus*). Left and right homology regions are indicated with striped blocks. “goi” refers to the gene of interest to be deleted. The arrow indicates the location of the XmaI sequence between the left and right homology regions. (B) DNA sequence of the intergenic region between *flaD* and *ATU0568*. Underlined are the stop and start codons respectively, as well as an alternative start codon for *ATU0568*. White dots show the insertion site of the transposon that landed in rightward orientation (referring to *kanR* transcription), while black dots indicate that the transposon landed in leftward orientation.
(TIF)

S6 Fig Motility assay of intergenic regions (IG1 and IG2). (A) Swim rings were imaged 48 h after inoculation. Shown are the averages of swim ring diameter for four replicates per strain in millimeters (mm) and standard deviation from the mean. Statistical analysis is shown in (B). (B) Different letters denote statistically significant differences (P < 0.05) according to Tukey multiple comparison test.
(TIF)

S7 Fig Quantification of swim ring diameter of suppressor analysis 3 days (A) and 6 days (B) post-inoculation. Swim rings from this suppressor analysis are shown in S7 Fig. Values are the averages of four replicates per strain. Error bars show standard deviation from the mean. Different letters denote statistically significant differences (P < 0.05) according to Tukey multiple comparison test.
(TIF)

S8 Fig Cell-cell hyper-adherence test. BB01 was modified to constitutively express *lacZ* from a synthetic transposon (*lacZ+*; blue colonies). This *lacZ+* strain and Δ*visNR* were grown as individual cultures in 5 ml of LB+Sm overnight at 30°C. Equal portions of these overnight cultures were mixed together, diluted, cultured for several hours, and plated on LB+Sm+X-Gal to test for cell hyper-adherence, which would have shown as sectored colonies.
(TIF)

S9 Fig Quantification of swim ring diameter of complementation tests for the *VisNR* knock-out mutants in Fig 6A. Shown are the averages of four swim rings per strain. Error bars show standard deviation from the mean. Different letters denote statistically significant differences (P < 0.05) according to Tukey multiple comparison test.
(TIF)

S1 Table Bacterial strains used in this study. (DOCX)

S2 Table Plasmids used in this study. (DOCX)

S3 Table Primers used in this study. (DOCX)

S1 File Disrupted genes with two or more independent transposon insertions. (XLSX)

S2 File Disrupted genes with single transposon insertions. (XLSX)

S3 File Annotated motility cluster of *Agrobacterium fabrum*. (XLSX)

S4 File Plasmid sequences. (DOCX)
