# Supplementary figures and images for: A large-scale genetic screen identifies genes essential for motility in Agrobacterium fabrum
Source: PLoS One. 2023 Jan 4;18(1):e0279936. doi: 10.1371/journal.pone.0279936 (PMC9812332; doi:10.1371/journal.pone.0279936)

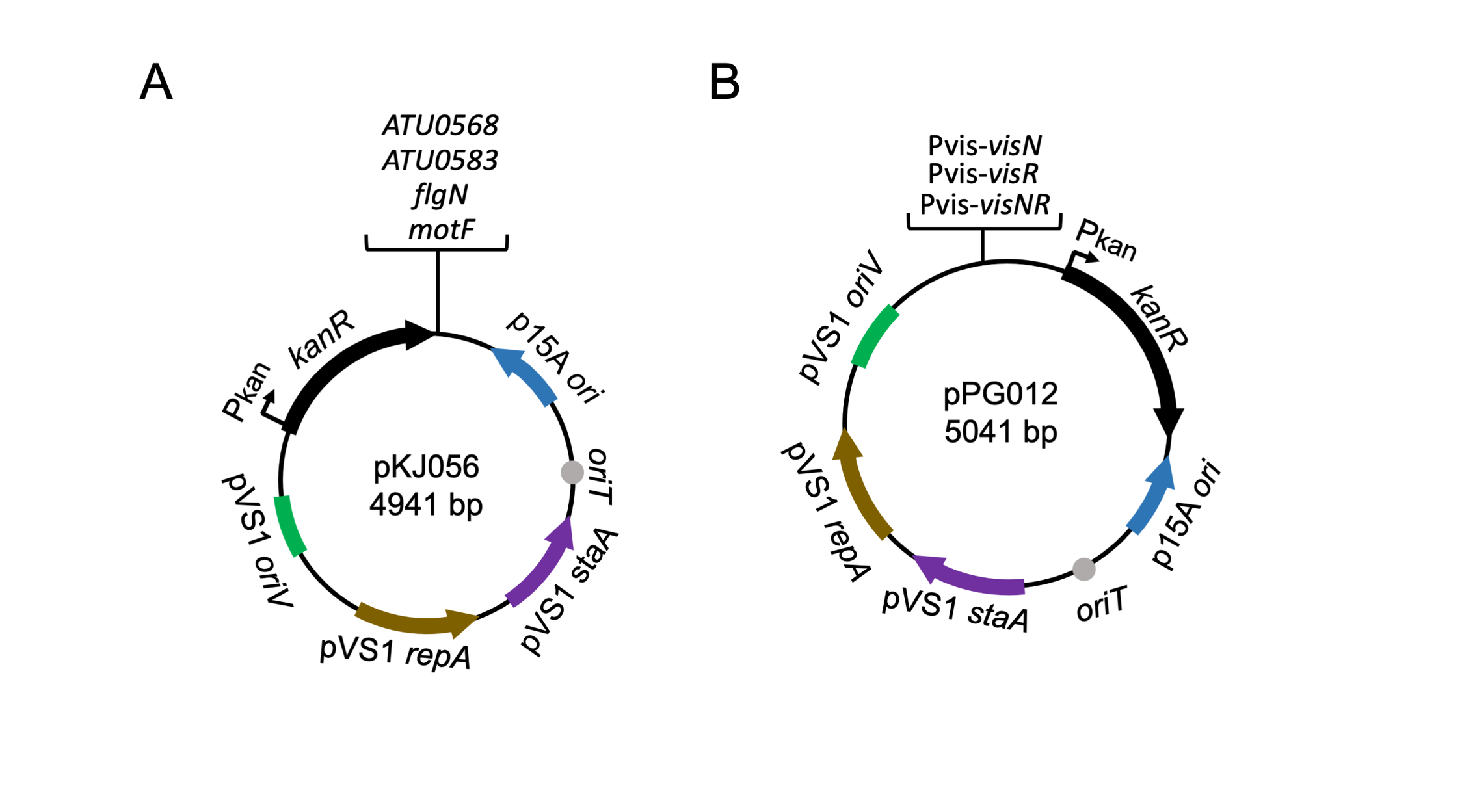

Supplement: S1 Fig — (A) Diagram of parent vector used to make complementation plasmids for ΔATU0568, ΔATU0583, ΔflgN, and ΔmotF strains. Each of the four genes were constitutively expressed by read-through transcription of the kanR gene. (B) Plasmid used as parent vector for visNR complementation derivates. visN, visR, and visNR labels show where each gene was inserted; transcription of visN, visR, and visNR is driven by the native Pvis promoter. (TIF) [file pone.0279936.s001.tif]

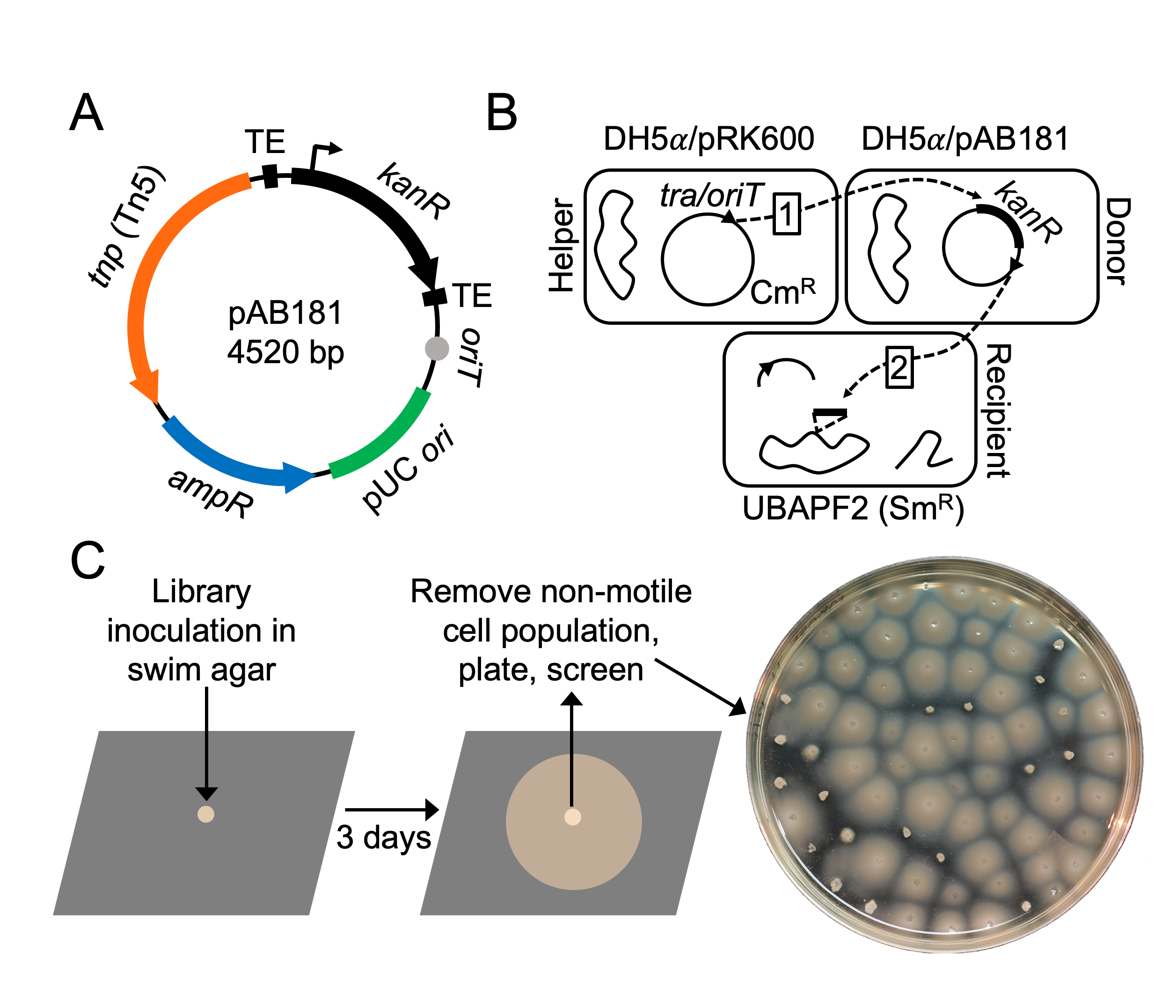

Supplement: S2 Fig — (A) Transposon delivery plasmid used to carry out the mutagenesis. TE labels show the location of the repeated transposon elements. (B) Triparental mating scheme involving helper plasmid pRK600 and A. fabrum recipient strain UBAPF2 (SmR). (C) Enrichment strategy enabling high-efficiency screening for motility mutations. (TIF) [file pone.0279936.s002.tif]

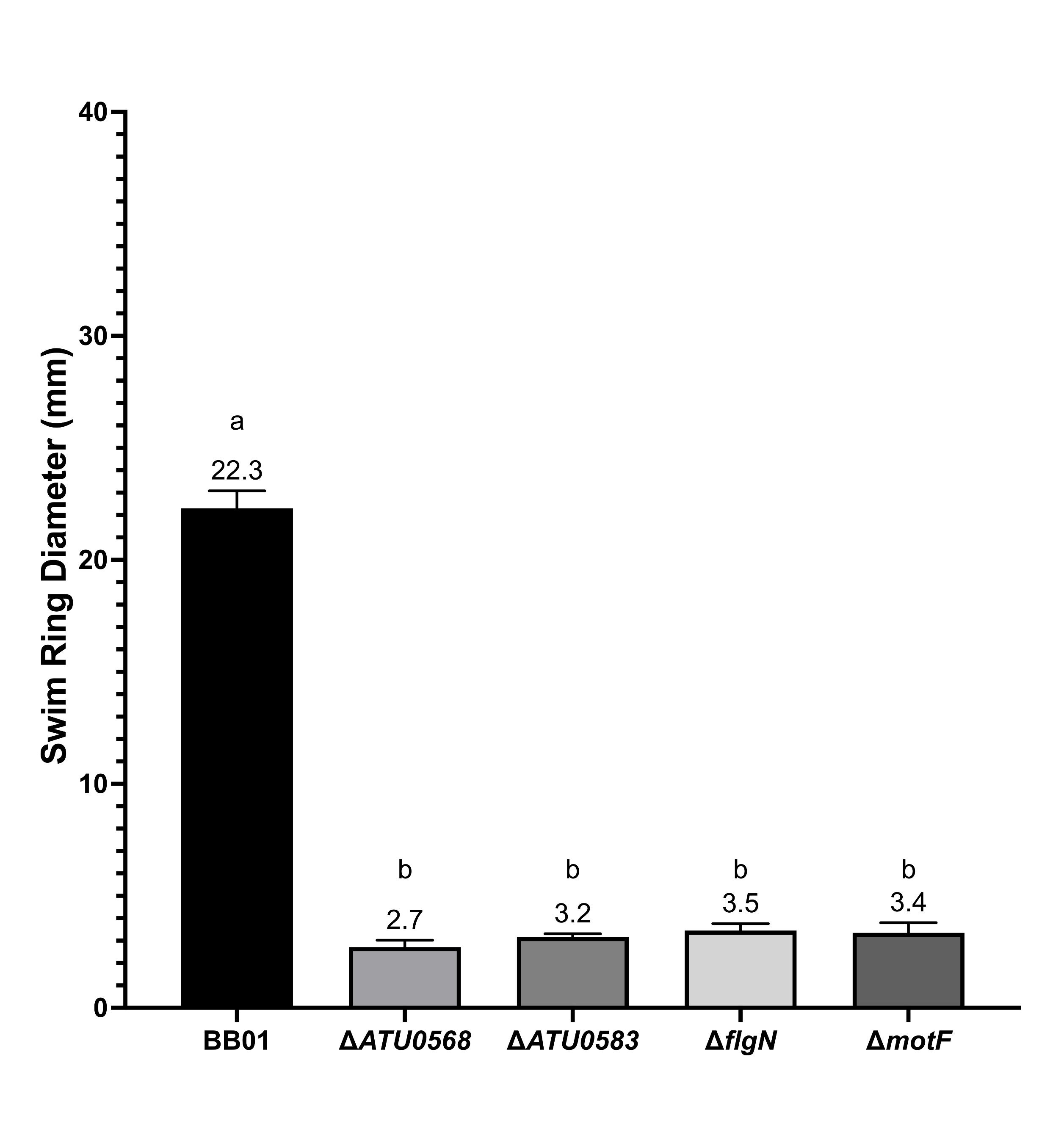

Supplement: S3 Fig — BB01 is the wild-type control. Values are the averages of four swim rings per strain. Error bars show standard deviation from the mean. Different letters denote statistically significant differences (P < 0.05) according to Tukey multiple comparison test. (TIF) [file pone.0279936.s003.tif]

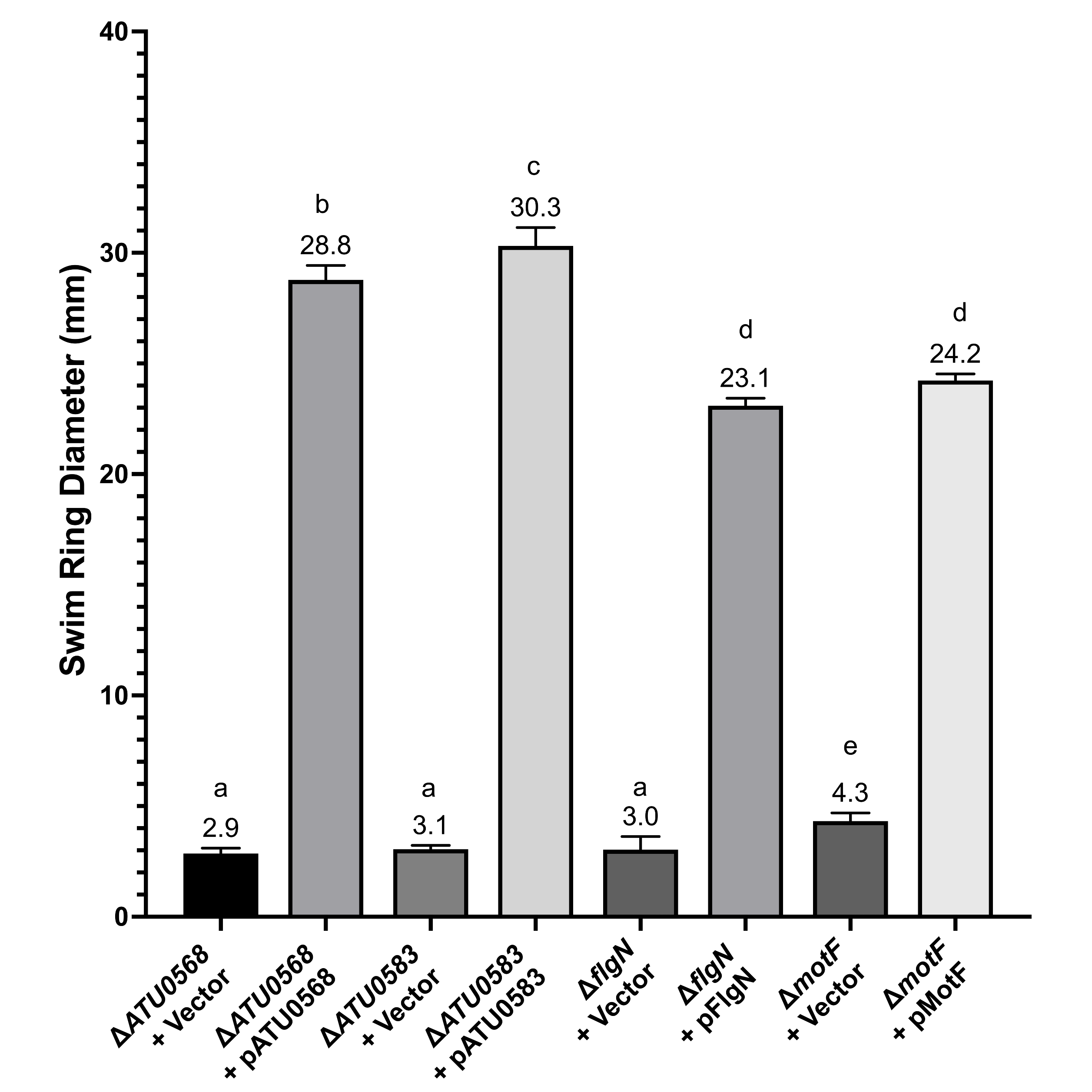

Supplement: S4 Fig — Values are the averages of four replicates per strain. Error bars show standard deviation from the mean. Different letters denote statistically significant differences (P < 0.05) according to Tukey multiple comparison test. (TIF) [file pone.0279936.s004.tif]

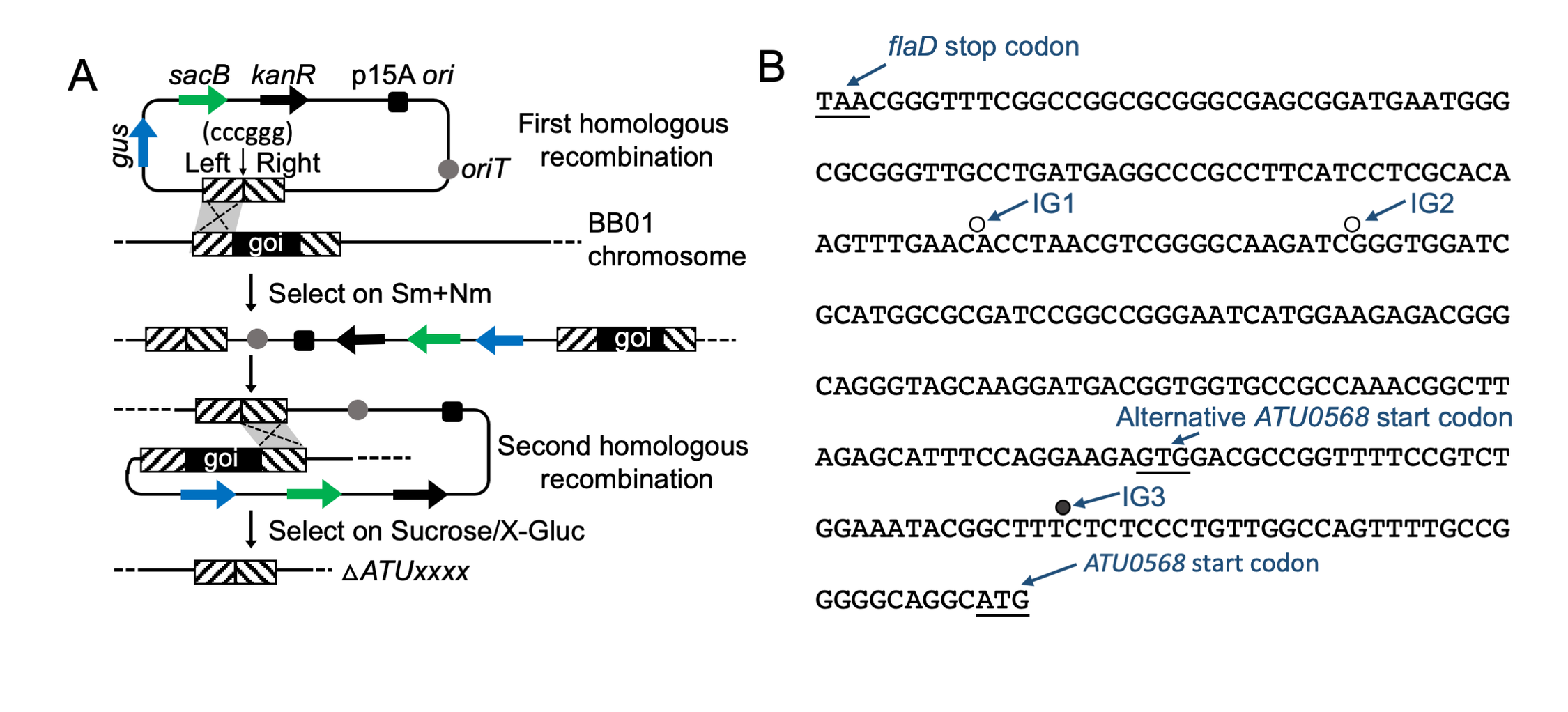

Supplement: S5 Fig — (A) The allelic replacement strategy used to construct deletion strains, using a plasmid that allows positive selection (kanR), negative selection (sacB), and color detection (gus). Left and right homology regions are indicated with striped blocks. “goi” refers to the gene of interest to be deleted. The arrow indicates the location of the XmaI sequence between the left and right homology regions. (B) DNA sequence of the intergenic region between flaD and ATU0568. Underlined are the stop and start codons respectively, as well as an alternative start codon for ATU0568. White dots show the insertion site of the transposon that landed in rightward orientation (referring to kanR transcription), while black dots indicate that the transposon landed in leftward orientation. (TIF) [file pone.0279936.s005.tif]

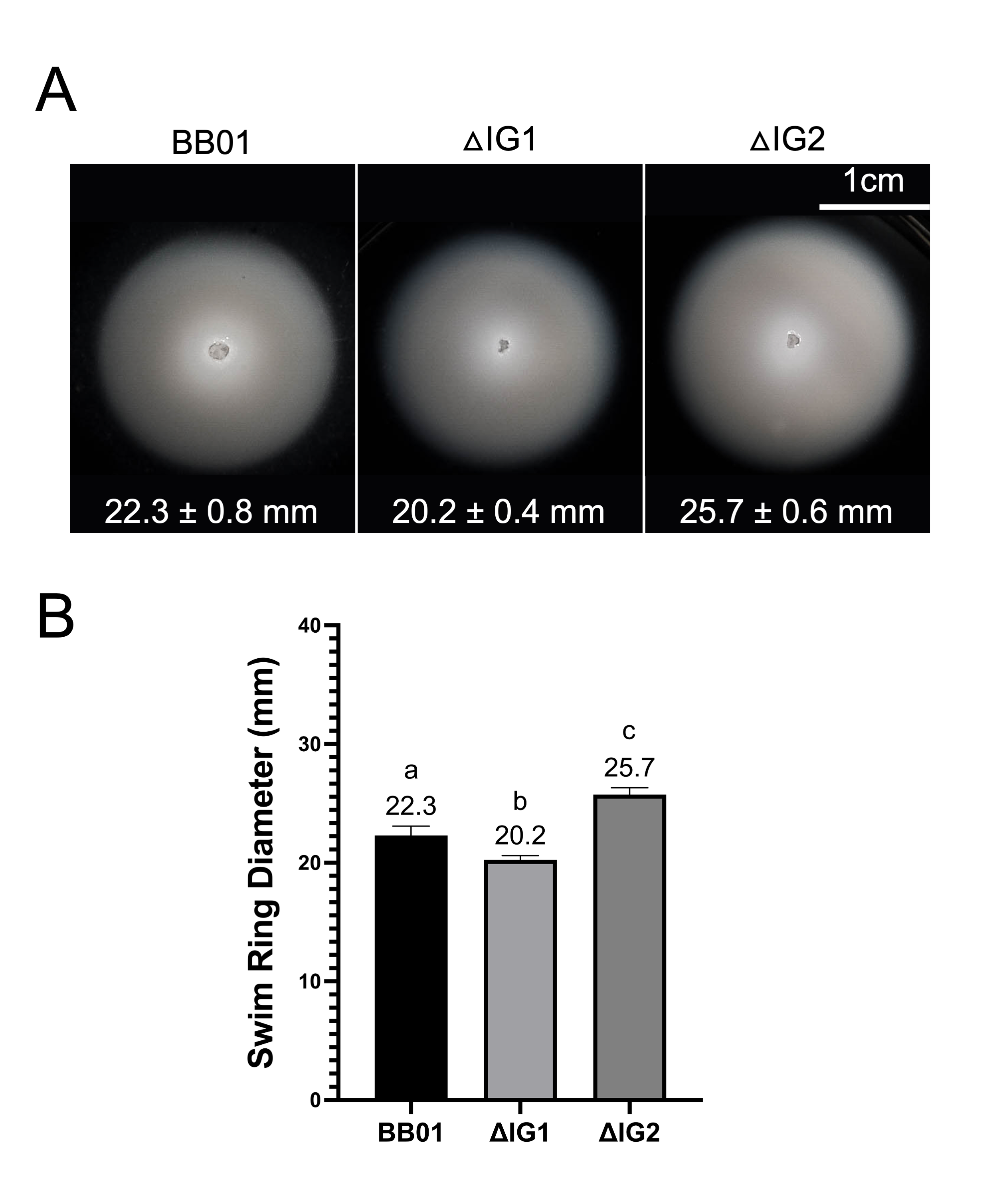

Supplement: S6 Fig — (A) Swim rings were imaged 48 h after inoculation. Shown are the averages of swim ring diameter for four replicates per strain in millimeters (mm) and standard deviation from the mean. Statistical analysis is shown in (B). (B) Different letters denote statistically significant differences (P < 0.05) according to Tukey multiple comparison test. (TIF) [file pone.0279936.s006.tif]

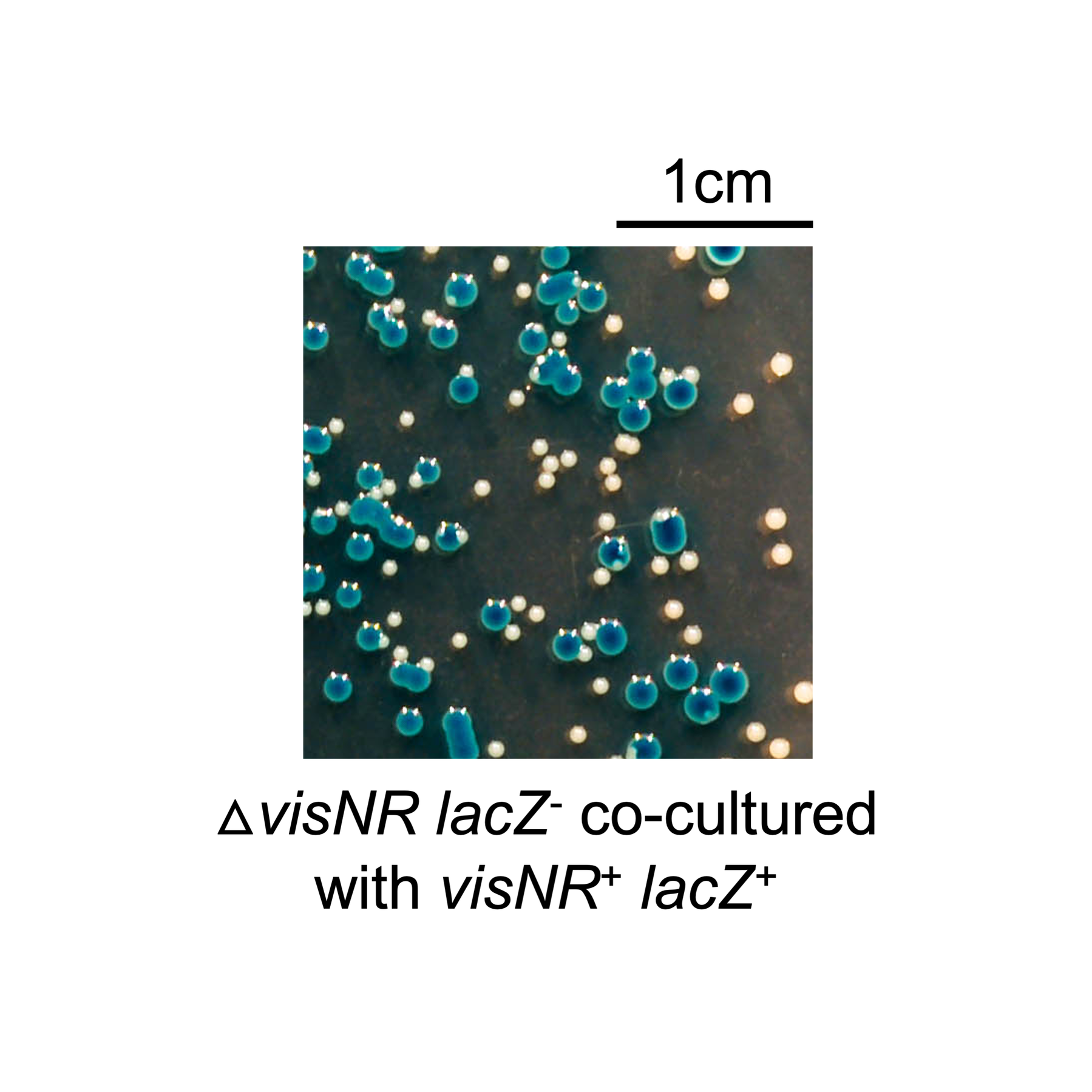

Supplement: S7 Fig — Swim rings from this suppressor analysis are shown in S7 Fig. Values are the averages of four replicates per strain. Error bars show standard deviation from the mean. Different letters denote statistically significant differences (P < 0.05) according to Tukey multiple comparison test. (TIF) [file pone.0279936.s007.tif]

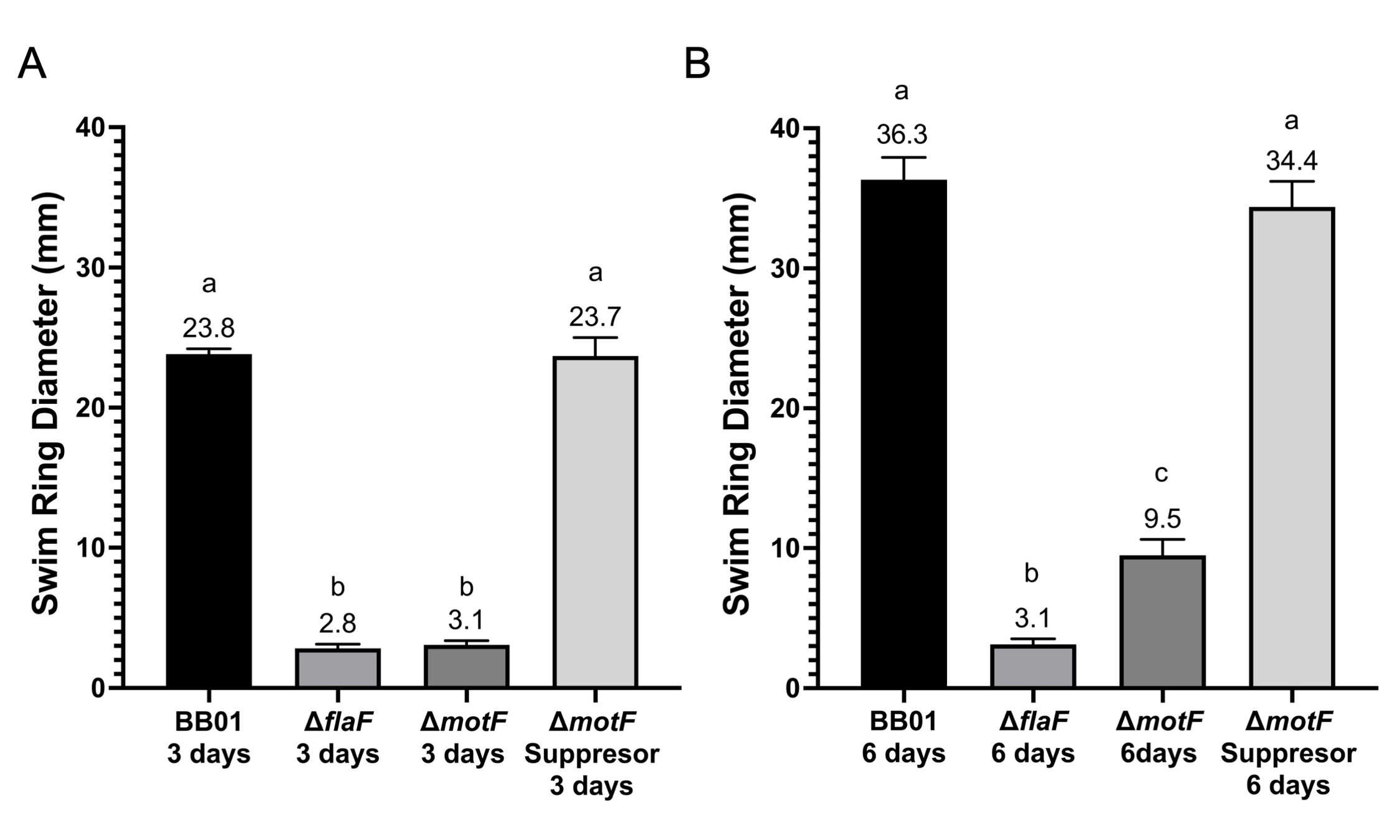

Supplement: S8 Fig — BB01 was modified to constitutively express lacZ from a synthetic transposon (lacZ+; blue colonies). This lacZ+ strain and ΔvisNR were grown as individual cultures in 5 ml of LB+Sm overnight at 30°C. Equal portions of these overnight cultures were mixed together, diluted, cultured for several hours, and plated on LB+Sm+X-Gal to test for cell hyper-adherence, which would have shown as sectored colonies. (TIF) [file pone.0279936.s008.tif]

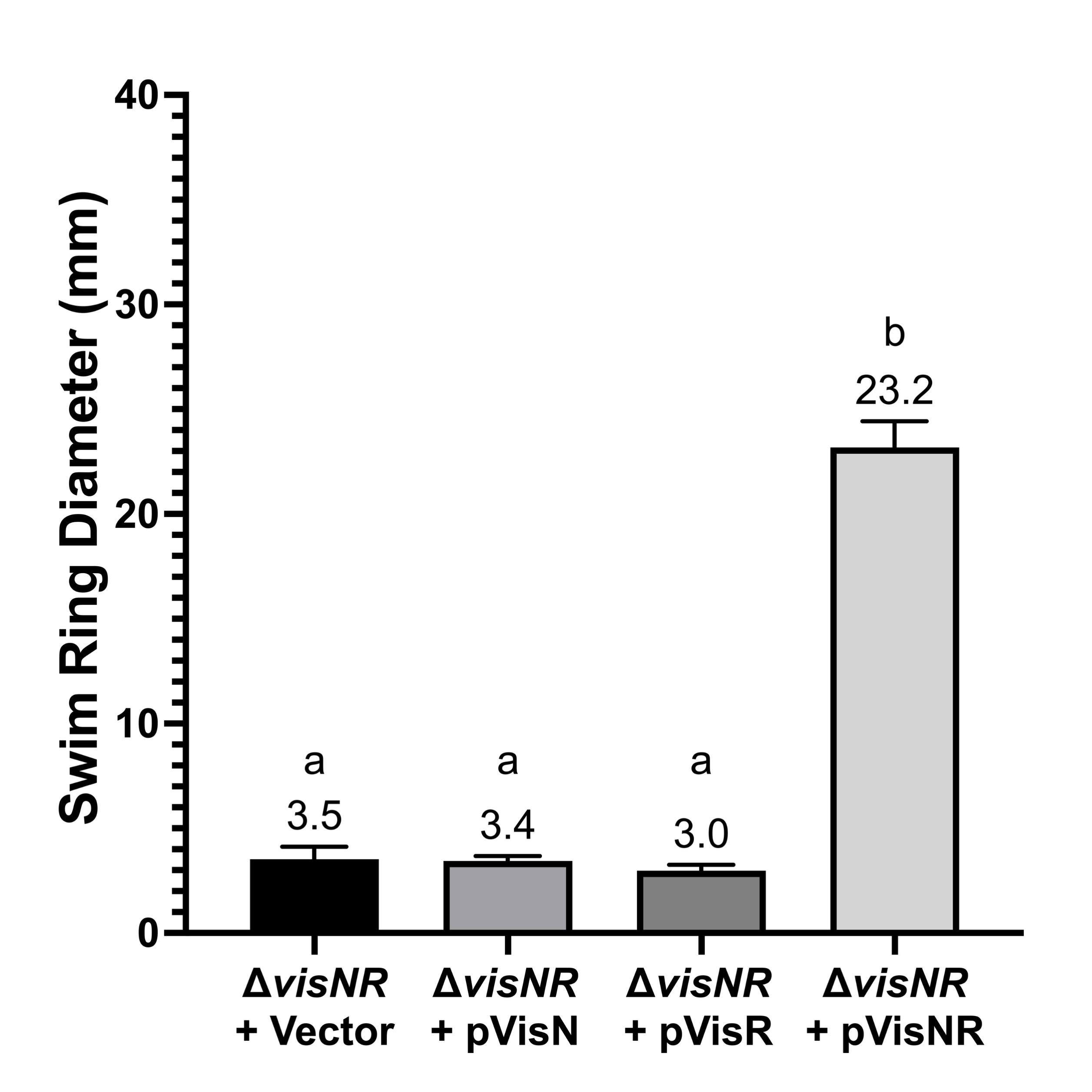

Supplement: S9 Fig — Shown are the averages of four swim rings per strain. Error bars show standard deviation from the mean. Different letters denote statistically significant differences (P < 0.05) according to Tukey multiple comparison test. (TIF) [file pone.0279936.s009.tif]
