# Supplementary material for: A large-scale genetic screen identifies genes essential for motility in Agrobacterium fabrum
Source: PLoS One. 2023 Jan 4;18(1):e0279936. doi: 10.1371/journal.pone.0279936 (PMC9812332; doi:10.1371/journal.pone.0279936)
Supplement: S1 Table — (DOCX) [file pone.0279936.s010.docx]

**Supporting Information for “A large-scale genetic screen identifies genes essential for motility in *Agrobacterium fabrum*”**

**S1 Table. Bacterial strains used in this study**

| **Strain name^[[1]](#footnote-1)^** | **Purpose/Description** | **Antibiotic Resistance** |
| --- | --- | --- |
| C237 | *Agrobacterium fabrum*. An isolate from UBAPF2 (plasmid cured derivative of C58)^[[2]](#footnote-2)^ |  |
| BB01 | SmR derivative of C237 | Sm |
| YS01 | SmR derivative of C237 | Sm |
| BM01 | SmR derivative of C237 | Sm |
| IW01 | SmR derivative of C237 | Sm |
| CI01 | SmR derivative of C237 | Sm |
| BL01 | SmR derivative of C237 | Sm |
| D223 | DH5α with transposon delivery vector pAB181 | Ap, Km |
| B001 | DH5α strain harboring helper plasmid pRK600^[[3]](#footnote-3)^ | Cm |
| D247 | Δ*ATU0524-0525(visNR)* derivative of BB01 | Sm |
| D249 | ΔIG1 derivative of BB01 | Sm |
| D250 | ΔIG2 derivative of BB01 | Sm |
| D251 | Δ*ATU0568* derivative of BB01 | Sm |
| D253 | Δ*ATU0577(flaF)* derivative of BB01 | Sm |
| D255 | Δ*ATU0583* derivative of BB01 | Sm |
| D256 | Δ*ATU0585(flgN)* derivative of BB01 | Sm |
| D257 | Δ*ATU8132* derivative of BB01 | Sm |
| D260 | Suppressor of Δ*ATU8132(motF),* derived from D257 | Sm |
| D265 | Δ*visNR* (D247) with pPG012 | Sm, Km |
| D266 | Δ*visNR* (D247) with pKJ121 | Sm, Km |
| D267 | Δ*visNR* (D247) with pKJ122 | Sm, Km |
| D268 | Δ*visNR* (D247) with pKJ120 | Sm, Km |
| KJ191 | Δ*ATU0568* (D251) with pKJ124 | Sm, Km |
| KJ193 | Δ*ATU0583* (D255) with pKJ126 | Sm, Km |
| KJ194 | Δ*ATU0585* (D256) with pKJ127 | Sm, Km |
| KJ196 | Δ*ATU8132* (D257) with pKJ129 | Sm, Km |
| KJ198 | Δ*ATU0568* (D251) with pKJ056 | Sm, Km |
| KJ199 | Δ*ATU0583* (D255) with pKJ056 | Sm, Km |
| KJ200 | Δ*ATU0585* (D256) with pKJ056 | Sm, Km |
| KJ201 | Δ*ATU8132* (D257) with pKJ056 | Sm, Km |

1. Except where noted below, all strains were created as part of this study. [↑](#footnote-ref-1)
2. Hynes MF, Simon R, Puhler A. The development of plasmid-free strains of Agrobacterium tumefaciens by using incompatibility with a Rhizobium meliloti plasmid to eliminate pAtC58. Plasmid. 1985;13(2):99-105. doi: 10.1016/0147-619x(85)90062-9. PubMed PMID: 4001194. [↑](#footnote-ref-2)
3. Griffitts JS, Carlyon RE, Erickson JH, Moulton JL, Barnett MJ, Toman CJ, et al. A Sinorhizobium meliloti osmosensory two-component system required for cyclic glucan export and symbiosis. Mol Microbiol. 2008;69(2):479-90. doi: 10.1111/j.1365-2958.2008.06304.x. PubMed PMID: 18630344. [↑](#footnote-ref-3)
