# Supplementary material for: A large-scale genetic screen identifies genes essential for motility in Agrobacterium fabrum
Source: PLoS One. 2023 Jan 4;18(1):e0279936. doi: 10.1371/journal.pone.0279936 (PMC9812332; doi:10.1371/journal.pone.0279936)
Supplement: S2 Table — (DOCX) [file pone.0279936.s011.docx]

**Supporting Information for “A large-scale genetic screen identifies genes essential for motility in *Agrobacterium fabrum*”**

**S2 Table. Plasmids used in this study**

| **Plasmid name** | **Purpose/Description** | **Antibiotic Resistance** |
| --- | --- | --- |
| pAB181 | Transposon delivery plasmid for mutagenesis | Ap, Km |
| pRK600 | Helper plasmid for mobilizing transposon delivery plasmid | Cm |
| pJG1108 | Parent plasmid for in-frame deletions (*p15Aori gus* *sacB* *kanR* *RK2oriT*) | Km |
| pJG1185 | pJG1108 derivative for deleting *ATU0568* | Km |
| pJG1187 | pJG1108 derivative for deleting *ATU0583* | Km |
| pJG1188 | pJG1108 derivative for deleting *ATU0585 (flgN)* | Km |
| pJG1189 | pJG1108 derivative for deleting *ATU8132 (motF)* | Km |
| pJG1190 | pJG1108 derivative for deleting *ATU0525-ATU0526 (visNR)* | Km |
| pJG1192 | pJG1108 derivative for deleting *ATU0577(flaF)* | Km |
| pJG1194 | pJG1108 derivative for deleting IG1 | Km |
| pJG1196 | pJG1108 derivative for deleting IG2 | Km |
| pPG012 | Parent plasmid for *visNR* complementation plasmids *(kanR p15Aori RK2oriT pVS1staA pVS1RepA pVS1ori)* | Km |
| pKJ056 | Parent plasmid for complementation plasmids: *ATU0568, ATU0583, flgN, motF* (*pV1ori pVS1repA pVS1staA* *p15Aori kanR* *RK2oriT*) | Km |
| pKJ120 | pPG012 derivative, *Pvis-visNR* (also known as pVisNR) | Km |
| pKJ121 | pPG012 derivative, *Pvis-visN* (also known as pVisN) | Km |
| pKJ122 | pPG012 derivative, *Pvis-visR* (also known as pVisR) | Km |
| pKJ124 | pKJ056 derivative for complementing *ATU0568* | Km |
| pKJ126 | pKJ056 derivative for complementing *ATU0583* | Km |
| pKJ127 | pKJ056 derivative for complementing *flgN* | Km |
| pKJ129 | pKJ056 derivative for complementing *motF* | Km |
