# Supplementary material for: A large-scale genetic screen identifies genes essential for motility in Agrobacterium fabrum
Source: PLoS One. 2023 Jan 4;18(1):e0279936. doi: 10.1371/journal.pone.0279936 (PMC9812332; doi:10.1371/journal.pone.0279936)
Supplement: S3 Table — (DOCX) [file pone.0279936.s012.docx]

**Supporting Information for “A large-scale genetic screen identifies genes essential for motility in *Agrobacterium fabrum*”**

**S3 Table. Primers used in this study**

| **Primer name** | **Sequence** | **Purpose/Description*** |
| --- | --- | --- |
| 2100 | AGCGCATCGCCTTC | First-round Arb-PCR forward |
| 2101 | CTATCGCCTTCTTGACGAG | Second-round Arb-PCR forward |
| 2102 | CCACGCGTCGACTAGTACNNNNNNNNNNACGCC | First-round Arb-PCR reverse (A) |
| 2103 | CCACGCGTCGACTAGTACNNNNNNNNNNTGCGG | First-round Arb-PCR reverse (B) |
| 2104 | CCACGCGTCGACTAGTAC | Second-round Arb-PCR reverse |
| CD07 | cgcTCTAGAgggcgagcggatgaatg | Left HR *ATU0568*-forward (for construction of plasmid pJG1185) |
| CD08 | TGCcccgggCTTgagaaatcgtgcaatcgacg | Left HR *ATU0568*-reverse (for construction of plasmid pJG1185) |
| CD09 | AAGcccgggGCAtccagtcttcgcaagg | Right HR *ATU0568*-forward (for construction of plasmid pJG1185) |
| CD10 | cgcGTCGAcctcttccttatcggcagg | Right HR *ATU0568*-reverse (for construction of plasmid pJG1185) |
| CD11 | CTCTTCAAGTAACGGGTTTCGG | PCR check *ATU0568*-forward |
| CD12 | GATGTTGGACTGGTCCTTCTTCTG | PCR check *ATU0568*-reverse |
| CD25 | cgcTCTAGAatgctgatcacgctgacg | Left HR *ATU0583*-forward (for construction of plasmid pJG1187) |
| CD26 | TGGcccgggGAGcttttccgagcgcttg | Left HR *ATU0583*-reverse (for construction of plasmid pJG1187) |
| CD27 | CTCcccgggCCAgcctccagcaagg | Right HR *ATU0583*-forward (for construction of plasmid pJG1187) |
| CD28 | cgcGTCGACtcgacggcagcaccaaag | Right HR *ATU0583*-reverse (for construction of plasmid pJG1187) |
| CD29 | CGCTCATCAGCGGACAG | PCR check *ATU0583*-forward |
| CD30 | GAGGAGGCACCGTCAAGC | PCR check *ATU0583*-reverse |
| CD37 | cgcTCTAgaaatctggaagagcatgatggc | Left HR *flgN*-forward (for construction of plasmid pJG1188) |
| CD38 | GGCcccgggGTTcgacataaggtcCATgcttg | Left HR *flgN*-reverse (for construction of plasmid pJG1188) |
| CD39 | AACcccgggGCCgatacggacggcac | Right HR *flgN*-forward (for construction of plasmid pJG1188) |
| CD40 | ccttGTCGACgcgcagC | Right HR *flgN*-reverse (for construction of plasmid pJG1188) |
| CD41 | GGAAGTTTACGGCAAGGGTAATG | PCR check *flgN*-forward |
| CD42 | GTTGCCGGCAGTTCGATCTT | PCR check *flgN*-reverse |
| CD43 | cgcTCTAGAgttcgacttcaaggtctccaatg | Left HR *motF*-forward (for construction of plasmid pJG1189) |
| CD44 | TGCcccgggCCAgacgccggtgagaag | Left HR *motF* -reverse (for construction of plasmid pJG1189) |
| CD45 | TGGcccgggGCAggagaggctgcgg | Right HR *motF* -forward (for construction of plasmid pJG1189) |
| CD46 | cgcGTCGAcgcgccattccagtcga | Right HR *motF* -reverse (for construction of plasmid pJG1189) |
| CD47 | GAGAACGACAATATCGGTAAGGACC | PCR check *motF*-forward |
| CD48 | CCTTGCCGCTGACCAGTT | PCR check *motF*-reverse |
| CD51 | cgctctagaGCCGTTTATGGATTGAACAGC | Left HR *visNR*-forward (for construction of plasmid pJG1190) |
| CD52 | TGCcccgggAAGCTGATCGCGCGGCAA | Left HR *visNR*-reverse (for construction of plasmid pJG1190) |
| CD53 | AAGcccgggAAGGCCATCAGGCTCGG | Right HR *visNR*-forward (for construction of plasmid pJG1190) |
| CD54 | cgcGTCGAcATGATGTTGTCCTTCAAGCG | Right HR *visNR*-reverse (for construction of plasmid pJG1190) |
| CD55 | GTTTGCTGGGAATTGGCTGAG | PCR check *visNR*-forward |
| CD56 | CGTGCGTATCACGCAGC | PCR check *visNR*-reverse |
| CD63 | cgctctagaCCATCAATGGTCTGAACCAGC | Left HR *flaF*-forward (for construction of plasmid pJG1192) |
| CD64 | TGCcccgggCATGATTTCGGCGTAGGAAAAC | Left HR *flaF* -reverse (for construction of plasmid pJG1192) |
| CD65 | AAGcccgggACAACCATCATCAGGGATGG | Right HR *flaF* -forward (for construction of plasmid pJG1192) |
| CD66 | cgcGTCGAcCATGCTGGAAGCAATTGAGC | Right HR *flaF*-reverse (for construction of plasmid pJG1192) |
| CD67 | GTCGGTGACGAATATCGGC | PCR check *flaF* -forward |
| CD68 | GCTTCAGTTCCGCCAGAATC | PCR check *flaF* -reverse |
| CD75 | cgctctagaCTTCCAGCCTCGGTTCG | Left HR IG1-forward (for construction of plasmid pJG1194) |
| CD76 | TGCcccgggAACTTGTGCGAGGATGAAGG | Left HR IG1-reverse (for construction of plasmid pJG1194) |
| CD77 | AAGcccgggACGTCGGGGCAAGATCG | Right HR IG1-forward (for construction of plasmid pJG1194) |
| CD78 | cgcGTCGAcAAACCAGAGGCGAAATCGG | Right HR IG1-reverse (for construction of plasmid pJG1194) |
| CD79 | CTCGAGGCAATGACCAGCAC | PCR check IG1-forward |
| CD80 | CGAAACCGGAGGTGATATCGG | PCR check IG1-reverse |
| CD81 | cgctctagaAGAGCCGTATCAGCATGCAG | Left HR IG2-forward (for construction of plasmid pJG1196) |
| CD82 | TGCcccgggTGCCCCGACGTTAGGTG | Left HR IG2-reverse (for construction of plasmid pJG1196) |
| CD83 | AAGcccgggGATCGCATGGCGCGATC | Right HR IG2-forward (for construction of plasmid pJG1196) |
| CD84 | cgcGTCGAcAAACCGGAGGTGATATCGGC | Right HR IG2-reverse (for construction of plasmid pJG1196) |
| CD85 | TTCCAGCCTCGGTTCGCT | PCR check IG2-forward |
| CD86 | CATCGACGGGTGTCAAATCG | PCR check IG2-reverse |
| oKJ392 | GCCAGGCAATCTACCAGG | PCR check complementation *visNR* plasmids-forward |
| oKJ393 | CCTCTCCACCCAAGCG | PCR check complementation *visNR* plasmids-reverse |
| oKJ396 | cgcGGATCCTGCGCAAATGGGC | Complementation plasmid *Pvis*-forward (for construction of plasmid pKJ120, pKJ121, pKJ122) |
| oKJ397 | gcgTCTAGAgcgtttgaccggcttaaggg | Complementation plasmid *visR*-reverse (for construction of plasmid pKJ120, pKJ122) |
| oKJ398 | gcgTCTAGAcgtctgtaccatccgtcc | Complementation plasmid *visN*-reverse (for construction of plasmid pKJ121) |
| oKJ399 | gcgCCCGGGcaagggcttacccatgcc | Complementation plasmid *Pvis*-reverse (for construction of plasmid pKJ122) |
| oKJ400 | gcgCCCGGGtcggaggcaattgcctacgcc | Complementation plasmid *visR*-forward (for construction of plasmid pKJ122) |
| oKJ410 | cgcGAATTCcctggtatatATGAGCGCGTCGATTGCAC | Complementation plasmid *P_kan_ATU0568*-forward (for construction of plasmid pKJ124) |
| oKJ411 | gcgGGATCCTCATTTCATCACCTTGCGAAG | Complementation plasmid *P_kan_ATU0568*-reverse (for construction of plasmid pKJ124) |
| oKJ413 | cgcGAATTCcctggtatatATGGCTTCGGACAAGCGC | Complementation plasmid *P_kan_ATU0583*-forward (for construction of plasmid pKJ126) |
| oKJ414 | cgcGGATCCCTATGGATCGCCAACCTTG | Complementation plasmid *P_kan_ATU0583*-reverse (for construction of plasmid pKJ126) |
| oKJ415 | cgcGAATTCggaggtatatATGGACCTTATGTCGAACGAC | Complementation plasmid *P_kan_flgN*-forward (for construction of plasmid pKJ127) |
| oKJ417 | gcgGGATCCtcaggaggctgcgccg | Complementation plasmid *P_kan_flgN*-reverse (for construction of plasmid pKJ127) |
| oKJ418 | cgcGAATTCggaggtatatATGTTGAAGCTTCTTCTCACC | Complementation plasmid *P_kan_motF*-forward (for construction of plasmid pKJ129) |
| oKJ420 | cgcGGATCCTCAGTGGCTGGGTGCC | Complementation plasmid *P_kan_motF*-reverse (for construction of plasmid pKJ129) |
| oKJ421 | GCTTCCTCGTGCTTTACGG | PCR check complementation plasmids-forward |
| oKJ422 | CCTGCCACATGAAGCACTTC | PCR check complementation plasmids-reverse |

*HR= Homology Region
